# Targeted Regulation of Pancreatic Islet Function by Natural Products Against Type 2 Diabetes Mellitus: Advances and Prospects

**DOI:** 10.3390/biom16091338

**Published:** 2026-09-15

**Authors:** Guanrong Qiao, Oluwaniyi Isaiah Adejobi, Xuefang Li, Yaqin Yang, Xudong He, Li Li, Yue Zhou, Jiawei Li, Hao Li, Fan Zhang, Jie Yu

**Affiliations:** Yunnan Key Laboratory of Southern Medicinal Utilization, College of Chinese Materia Medica, Yunnan University of Chinese Medicine, 1076 Yuhua Road, Kunming 650500, China; qiaoguanrong@ynucm.edu.cn (G.Q.); nheyoh@gmail.com (O.I.A.); lixuefang@ynucm.edu.cn (X.L.); rc.yaqin0902@zcmu.edu.cn (Y.Y.); hexudong@ynucm.edu.cn (X.H.); lili@ynucm.edu.cn (L.L.); zhouyue@ynucm.edu.cn (Y.Z.); lijiawei@ynucm.edu.cn (J.L.); lihao2@metware.cn (H.L.)

**Keywords:** natural products, phytocompound, type 2 diabetes mellitus, pancreatic β-cells, islet dysfunction

## Abstract

Type 2 diabetes mellitus (T2DM) is a major global health challenge, with insulin resistance (IR) and islet dysfunction as its core pathophysiology. Current glucose-lowering agents, including insulin secretagogues, insulin sensitizers, DPP–IV inhibitors, SGLT-2 inhibitors, and GLP-1 receptor agonists, effectively control blood glucose and may partly improve β-cell function indirectly, but they do not directly target the functional defects of pancreatic β cells. Developing drugs that precisely modulate islet function is therefore a key direction. Natural products, with their structural diversity, established glucose-lowering activity, and derivation from medicinal plants with long clinical use, have become an important source of lead compounds for drugs targeting these mechanisms. The main pathways through which natural products act include: (1) promoting insulin secretion through direct and indirect secretagogue mechanisms (modulating ion channels, metabolic enzymes, incretin signaling); (2) preserving β-cell function and mass by maintaining the differentiated phenotype, promoting regeneration, alleviating oxidative stress, inhibiting apoptosis and pyroptosis, attenuating inflammation, and suppressing hIAPP toxic aggregation and endoplasmic reticulum stress; and (3) other pathways with incompletely elucidated mechanisms that also contribute to islet protection. This article delineates these molecular mechanisms and highlights the limited ability of current agents to directly restore and protect β-cell function. It provides a theoretical basis for developing novel drugs that integrate glycemic control with islet restoration based on natural lead compounds, and offers a framework for further mechanistic studies. In addition, it encourages the exploration of unidentified active constituents and unique pathways, while also addressing challenges such as target identification and structure–activity relationships.

## 1. Introduction

Diabetes mellitus has emerged as a major global public health challenge. According to the report *World health statistics 2025: monitoring health for the Sustainable Development Goals* released by the World Health Organization in 2025, although global healthy life expectancy increased from 58.1 to 63.5 years between 2000 and 2019, the burden of premature mortality caused by noncommunicable diseases (NCDs) remains substantial: in 2021, approximately 18 million people under the age of 70 died from NCDs, accounting for more than 50% of all deaths in this age group. Among these, diabetes is an important contributing factor. In the population aged 30 years and older, the increasing disease burden associated with diabetes has reduced global healthy life expectancy by approximately 0.14 years, making it one of the leading causes of premature death [1]. Since 2011, China has had the largest number of people with diabetes worldwide, and its direct medical expenditure on diabetes ranks second globally. Complications such as cardiovascular disease and chronic kidney disease are the primary drivers of rising healthcare costs [2]. Moreover, diabetes complications themselves impose a heavy health and socioeconomic burden, exemplified by blindness resulting from diabetic retinopathy, the high prevalence of diabetic kidney disease, and the high incidence of diabetes-related cardiovascular events. Among all individuals with diabetes, T2DM accounts for 90–95% of cases [3].

**Figure 1:**
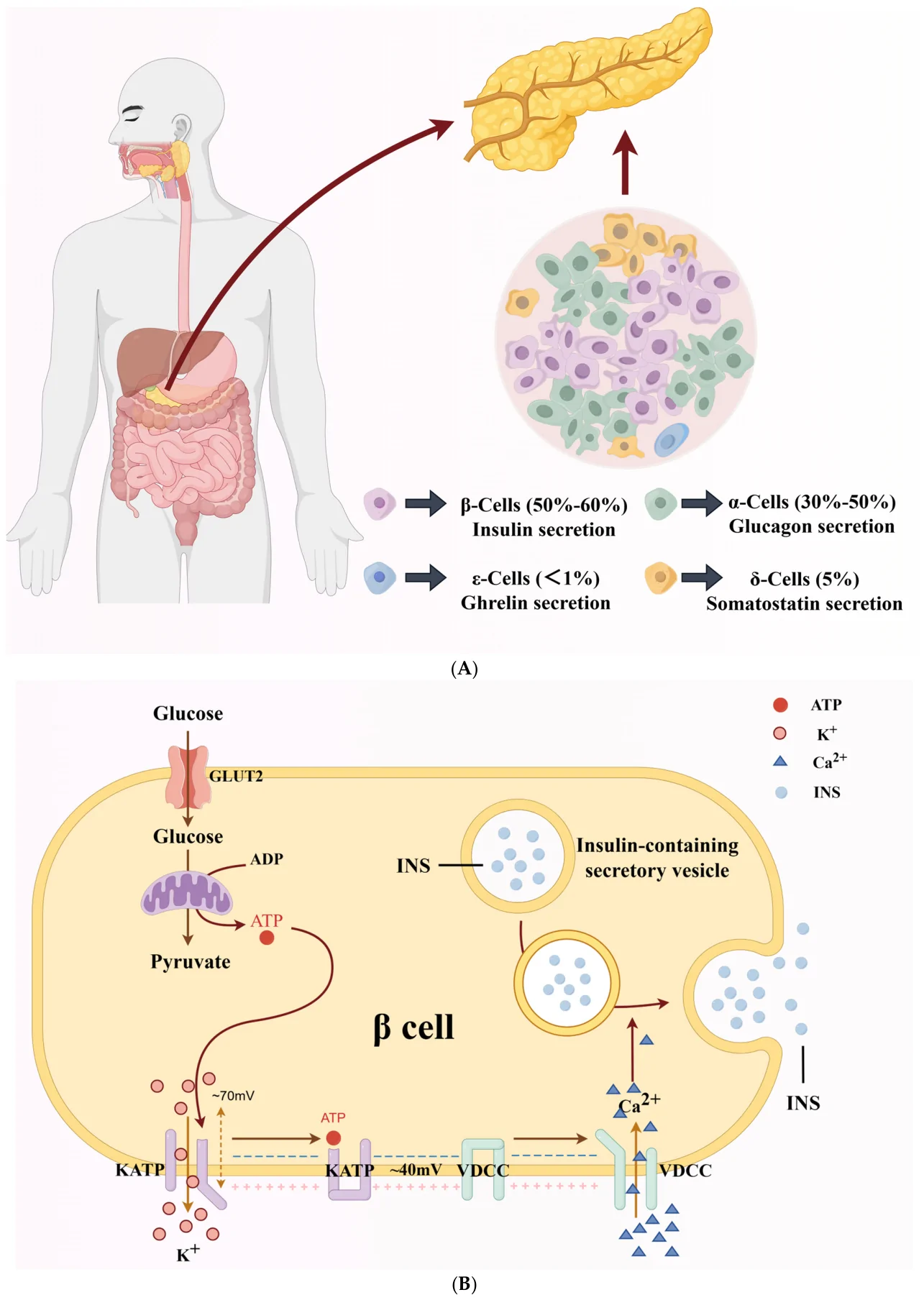
Islet structure (**A**) and GSIS process (**B**). (**A**) Islet endocrine cell composition. The islet contains four main endocrine cell types: α, β, δ, and ε cells. α cells secrete glucagon and comprise approximately 30–50% of total islet cells; β cells secrete insulin and are the most abundant, comprising approximately 50–60%; δ cells secrete somatostatin and account for approximately 5%; ε cells secrete ghrelin and are the least numerous, accounting for less than 1%. (**B**) GSIS. After glucose enters pancreatic β cells, metabolic pathways including the TCA cycle elevate the intracellular ATP/ADP ratio, promoting closure of KATP channels on the plasma membrane, which triggers membrane depolarization and subsequent opening of VDCCs, leading to a Ca^2+^ influx. The increase in intracellular Ca^2+^ concentration drives the fusion of insulin-containing vesicles with the plasma membrane, releasing insulin by exocytosis. This KATP-VDCC signaling axis represents a key pathway through which natural products promote insulin secretion. INS: Insulin; KATP: ATP-sensitive potassium channel; VDCC: voltage-dependent calcium channel. All images were produced using Figdraw.

T2DM is both a key pathological outcome and a core component of metabolic syndrome (MetS). Its pathogenesis is not driven solely by hyperglycemia but rather arises from the interplay of multiple metabolic disturbances (e.g., obesity and dyslipidemia) within the MetS framework. These disturbances converge through shared molecular pathways, including oxidative stress and chronic inflammation, to promote IR and islet dysfunction, which together constitute the cornerstone of T2DM pathogenesis [4,5]. Therefore, islet dysfunction is closely linked to both the pathogenesis and therapeutic intervention of T2DM. The pancreas is a complex organ with both exocrine and endocrine functions, and the precise regulation of islet function is a core element influencing the pathological progression of T2DM. At the anatomical and functional levels, although the islets are a component of the pancreas, they are fundamentally distinct from the exocrine pancreas in terms of tissue morphology and physiological function: the exocrine pancreas is composed of acinar and ductal systems responsible for the synthesis and secretion of digestive enzymes, whereas the endocrine pancreas consists of islets scattered within the exocrine tissue. The precise modulation of islet function relies on a highly heterogeneous cell population comprising insulin-secreting β cells, glucagon-secreting α cells, somatostatin-secreting δ cells, and ghrelin-secreting ε cells (Figure 1A). These cells operate in a coordinated manner through complex communication networks: β cells govern insulin secretion, α cells exert antagonistic effects to regulate blood glucose levels, and δ cells provide local fine-tuning via paracrine signaling. At the same time, the functional heterogeneity within the β-cell population further ensures the precision of glucose sensing and insulin release [4]. Upon entering circulation, the hormones secreted by these cells act on specific receptors in target organs such as the liver, muscle, and adipose tissue, thereby not only coordinating pancreatic exocrine and endocrine functions but also playing a central role in systemic energy metabolism and the maintenance of cellular homeostasis [6]. β cells are among the most important cell types in the islet, and glucose-stimulated insulin secretion (GSIS) is the core function by which pancreatic β cells rapidly respond to blood glucose fluctuations. The molecular basis is as follows: after glucose enters the β cell, its metabolism increases the intracellular ATP/ADP ratio, which causes the closure of ATP-sensitive potassium channels (KATP channels) on the plasma membrane, triggering membrane depolarization. This in turn opens voltage-dependent calcium channels (VDCCs), triggering a Ca^2+^ influx. The transient increase in intracellular Ca^2+^ concentration serves as the key triggering signal that drives the fusion of insulin-containing vesicles with the plasma membrane and the exocytotic release of insulin. This exocytotic process mediated by the KATP–VDCC pathway represents the final effector step by which natural products promote insulin secretion (Figure 1B).

Compared with existing reviews on natural products and pancreatic β-cell function, the present review focuses specifically on the direct modulation of β-cell function and survival under conditions relevant to chronic T2DM progression. In particular, we emphasize mechanisms such as toxic hIAPP aggregation, NLRP3 inflammasome-mediated pyroptosis, and endoplasmic reticulum stress, which have received limited systematic attention in earlier reviews (Table 1).

Currently, glucose-lowering agents in clinical use include both oral and injectable formulations. Oral agents comprise insulin secretagogues, biguanides, insulin sensitizers, DPP–IV inhibitors, and SGLT-2 inhibitors, whereas injectable agents mainly include insulin, insulin analogs, and GLP-1 receptor agonists. Contemporary T2DM management has moved from simple glucose lowering toward comprehensive organ protection and medication safety. Among these therapies, incretin-based agents, particularly GLP-1 receptor agonists and DPP–IV inhibitors, improve glucose-dependent insulin secretion and may provide indirect β-cell benefits through better glycemic control and reduced glucotoxicity and lipotoxicity [10]. However, an ideal drug that specifically and durably improves β-cell function with a favorable safety profile is still lacking because current agents do not directly reverse intrinsic β-cell defects such as impaired proliferative capacity, increased apoptotic susceptibility, or oxidative damage [5,11,12]. In addition, certain insulin secretagogues, despite effective glucose lowering, may further increase the burden on already stressed β cells [13]. Against this background, natural active ingredients from diverse sources have attracted increasing attention because of their glucose-lowering potential and multi-target pharmacological characteristics. Numerous preclinical studies have demonstrated that various natural products directly modulate islet function by promoting insulin secretion and enhancing β-cell survival and function [14,15,16,17]. In parallel, their anti-inflammatory and antioxidant activities may indirectly contribute to the long-term maintenance of islet function. These properties support the development of natural products as targeted islet-function modulators and as a complementary strategy to current therapies.

## 2. Islet Dysfunction Is the Core Pathophysiological Basis of T2DM

Although substantial individual differences exist among patients with T2DM, the core pathological mechanism in the majority of cases can still be attributed to hyperglycemia, driven jointly by IR and secondary β-cell dysfunction [5]. Persistent hyperglycemia not only further exacerbates insulin resistance but also directly impairs β-cell function, thereby forming a vicious cycle. Hyperglycemia often coexists with metabolic disturbances such as dyslipidemia and elevated inflammatory cytokines, which exacerbate these pathological changes through pathways involving inflammation, endoplasmic reticulum stress, and oxidative stress [4,5]. During this process, β cells not only exhibit impaired insulin secretion but also gradually decrease in absolute number with disease progression, ultimately leading to a continuous decline in the compensatory capacity of β-cell function [18].

Therefore, preserving and restoring pancreatic β-cell function and mass, thereby interrupting this vicious cycle, has become one of the core strategies for T2DM treatment. The precise regulation of islet function fundamentally depends on the accurate sensing of glucose by β cells and their capacity for insulin secretion. Glucose is not only the primary signal triggering insulin secretion but also a substrate for the amplifying effects of insulin secretagogues such as incretins [18]. The critical turning point in the progression from insulin resistance to clinical diabetes is precisely the decompensation of β-cell function. Accordingly, current research is increasingly focused on identifying interventions that can directly protect β cells, enhance glucose-stimulated insulin secretion, and maintain their survival.

## 3. Natural Active Ingredients Regulate Islet Function Through Multi-Target Mechanisms of Action

**Figure 2:**
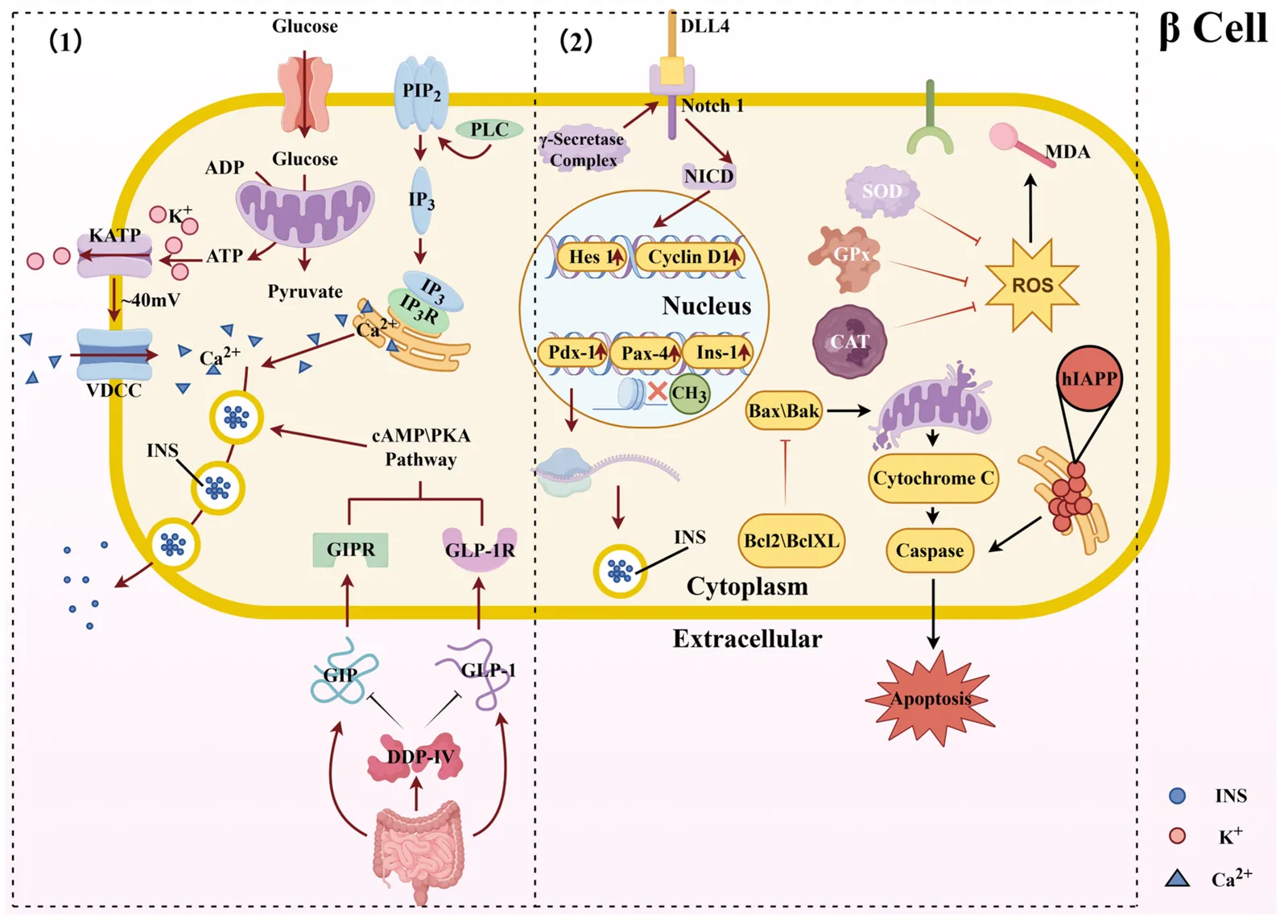
Core dimensions of natural products in regulating pancreatic β-cell function. Natural products regulate β-cell function through two core dimensions: (**1**) promoting insulin secretion, via direct actions on β-cell ion channels and metabolic enzymes and indirect modulation of the incretin system, and (**2**) protecting pancreatic β-cell function and mass and improving islet homeostasis, encompassing maintenance of the differentiated β-cell phenotype and promotion of regeneration, alleviation of oxidative stress, inhibition of apoptosis and pyroptosis, and attenuation of inflammation. Abbreviations: PLC: Phospholipase C, PIP_2_: Phosphatidylinositol 4,5-bisphosphate, IP_3_: Inositol trisphosphate, IP_3_R: IP_3_ receptor, GIP: Glucose-dependent Insulinotropic Polypeptide, GIPR: Glucose-dependent Insulinotropic Polypeptide Receptor, GLP-1: Glucagon-Like Peptide-1, GLP-1R: Glucagon-Like Peptide-1 Receptor, DPP–IV: Dipeptidyl Peptidase-IV, cAMP: Cyclic adenosine monophosphate, PKA: Protein Kinase A, Pdx-1: Pancreatic and Duodenal Homeobox 1, DLL4: Delta-like ligand 4, Notch1: Notch Receptor 1, NICD: Notch Intracellular Domain, Hes1: Hes Family BHLH Transcription Factor 1, Pax-4: Paired box 4, ROS: Reactive Oxygen Species, MDA: Malondialdehyde, SOD: Superoxide Dismutase, GPx: Glutathione Peroxidase, CAT: Catalase, Bcl-2: B-cell lymphoma 2, Bcl-XL: B-cell lymphoma-extra large, Bax: BCL-2-associated X protein, Bak: BCL2 Antagonist/Killer, hIAPP: Islet amyloid polypeptide. All images were produced using Figdraw.

In multiple traditional medical systems, medicinal plants from diverse sources have long been used for the prevention and treatment of T2DM. These medicinal plants and the natural active ingredients they contain, by virtue of their unique chemical structures, provide a rich source of lead compounds for identifying key pharmacophores and designing novel T2DM therapeutic agents. Existing studies have demonstrated that these active ingredients can act on different molecular targets and signaling pathways, collectively achieving multi-dimensional regulation of islet function. This review will elucidate their mechanisms from the following core dimensions of action: (1) promoting insulin secretion from islets, involving multiple pathways such as directly acting on β-cell ion channels and metabolic enzymes and indirectly modulating the incretin system; (2) protecting pancreatic β-cell function and mass and improving islet homeostasis, encompassing multiple protective aspects including maintaining the differentiated phenotype of β cells and promoting regeneration, alleviating oxidative stress, inhibiting apoptosis and pyroptosis, and attenuating inflammation; and (3) other regulatory pathways with confirmed efficacy but not yet fully elucidated mechanisms. These mechanisms are interconnected and together form a multi-dimensional regulatory network through which natural products modulate islet function (Figure 2).

### 3.1. Promotion of Insulin Secretion from Islets

Impaired insulin secretory function of pancreatic β cells is one of the core pathophysiological events in the development and progression of T2DM. A large body of research has demonstrated that a variety of natural products can act on β cells through direct or indirect pathways to enhance GSIS. Based on their primary targets and signaling pathways, the relevant mechanisms can be categorized into four types: targeting KATP channels and VDCCs to trigger an extracellular Ca^2+^ influx, mobilizing endoplasmic reticulum calcium stores to elevate cytosolic Ca^2+^ concentration, directly enhancing secretion via other signaling pathways, and indirectly amplifying glucose-dependent insulinotropic signals by modulating the incretin system. These four categories of mechanisms will be discussed sequentially below.

#### 3.1.1. Targeting KATP Channels and VDCCs

**Figure 3:**
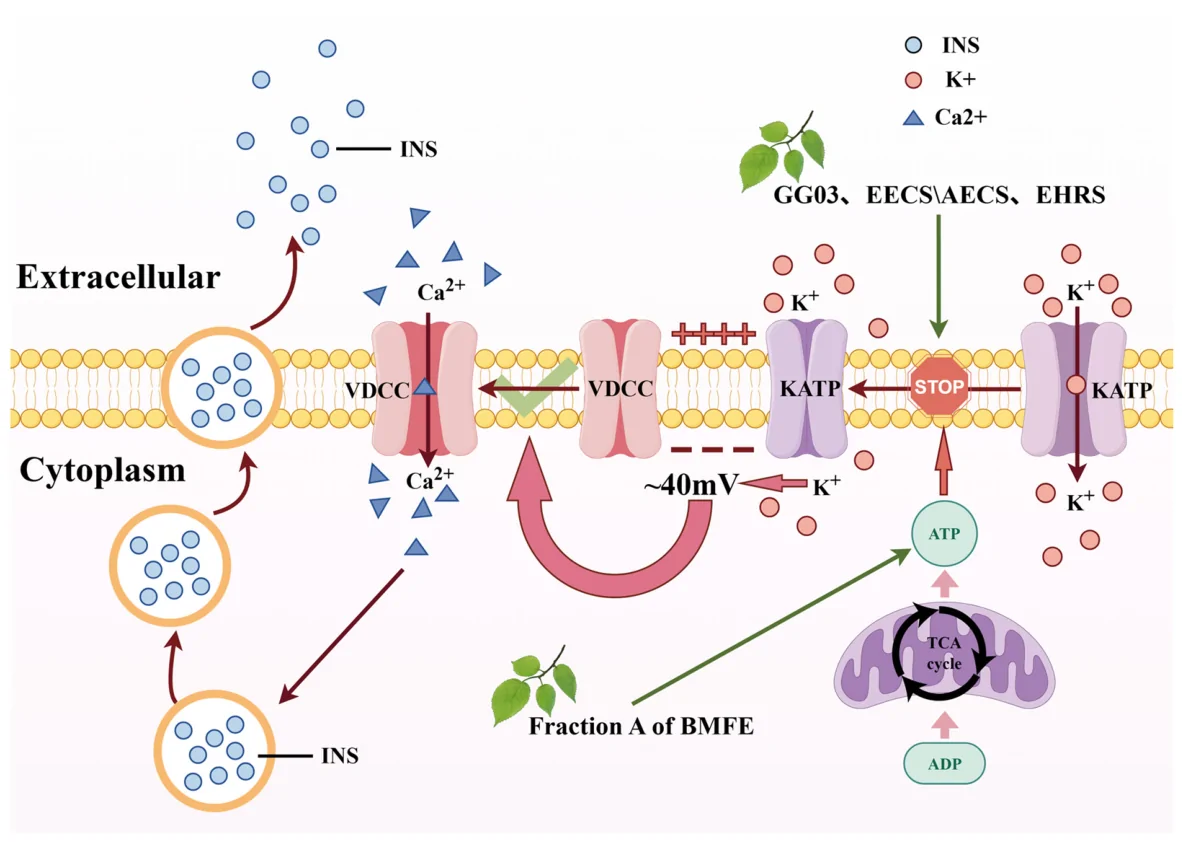
Natural products promote insulin secretion through the KATP–VDCC pathway. GG03, EECS, AECS, and EHRS can all close KATP channels, trigger membrane depolarization, and subsequently open VDCCs, triggering a Ca^2+^ influx; the hydrophobic fraction (Fraction A) of BMFE indirectly inhibits KATP channels by enhancing ATP production in β cells, thereby activating VDCCs and leading Ca^2+^ influx. Abbreviations: GG03: extract of *Zingiber officinale* Roscoe; EECS: ethanolic extract of Camellia sinensis; AECS: aqueous extract of *Camellia sinensis*; EHRS: ethanolic extract of leaves of *H. rosa-sinensis* L.; Fraction A of BMFE: hydrophobic fraction of bitter melon fruit extract. All images were produced using Figdraw.

Studies have demonstrated that a variety of natural products can promote insulin secretion by modulating GSIS (Figure 3). For example, the extract GG03 from *Zingiber officinale* Roscoe can close KATP channels, triggering membrane depolarization and a Ca^2+^ influx, thereby stimulating insulin secretion in alloxan-induced zebrafish larvae and ICR mice [19]. The ethanolic extract of *Camellia sinensis* (EECS) and the aqueous extract of *Camellia sinensis* (AECS) have been reported to improve islet function [20,21]; some studies have shown that EECS can directly promote insulin release by inhibiting KATP channels, inducing membrane depolarization, and elevating intracellular Ca^2+^ concentration in BRIN–BD11 cells and isolated mouse islets [22]. The ethanolic extract of leaves of *H. rosa-sinensis* L. (EHRS), a plant distributed in tropical regions of Asia, has been reported to possess antidiabetic activity [23,24] and similarly stimulates insulin secretion directly by closing KATP channels, triggering membrane depolarization and a Ca^2+^ influx in BRIN–BD11 cells and isolated rat islets [25]. Furthermore, bitter melon (*Momordica charantia* L.), a traditional Chinese medicine rich in multiple bioactive constituents [26,27], contains a hydrophobic fraction (Fraction A) in its fruit extract (bitter melon fruit extract, BMFE) which can enhance ATP production in β cells, subsequently inhibit KATP channels, activate VDCCs, and ultimately promote insulin secretion in INS-1D cells and isolated rat islets [28].

#### 3.1.2. IP_3_ Receptor-Mediated Mobilization of Endoplasmic Reticulum Calcium Stores

**Figure 4:**
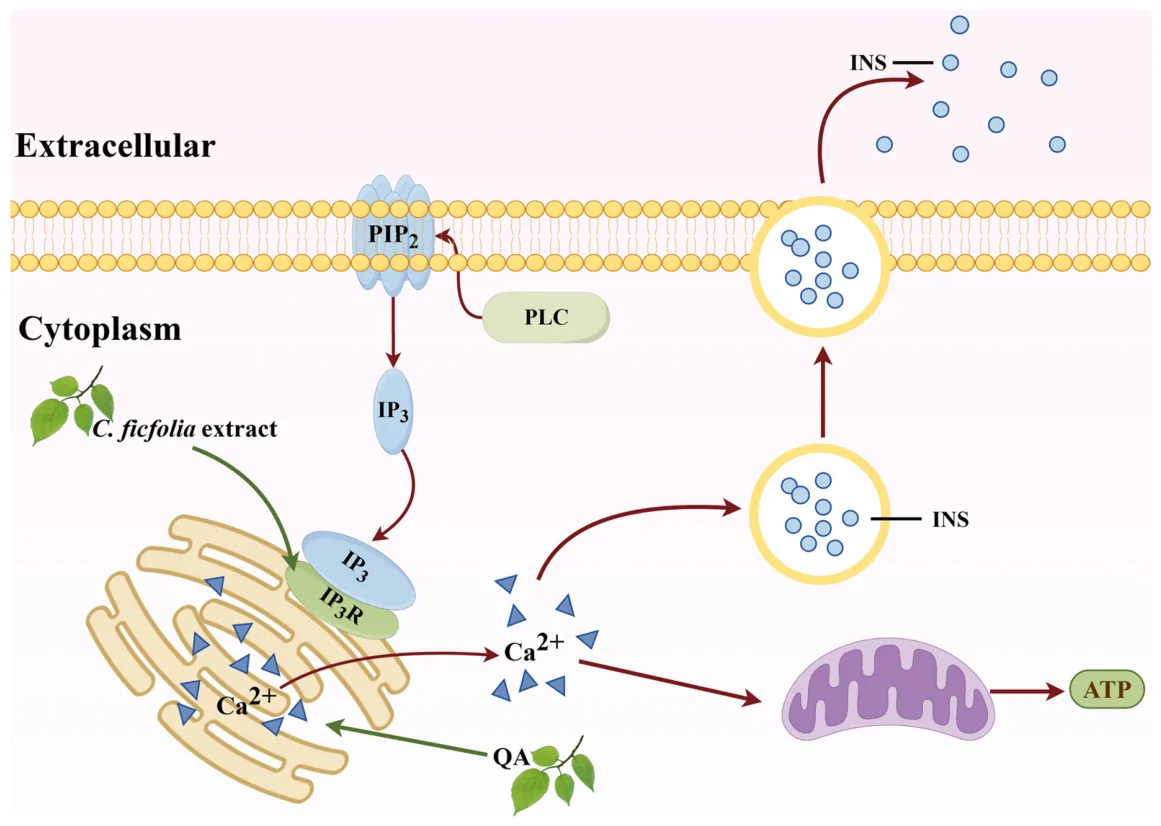
Natural products mobilize endoplasmic reticulum calcium stores to promote insulin secretion via the IP3 pathway. PLC hydrolyzes PIP_2_ to generate IP_3_, which binds to IP_3_ receptors on the endoplasmic reticulum membrane, triggering Ca^2+^ release from ER calcium stores and initiating INS exocytosis. *C. ficifolia* extract prompts Ca^2+^ release from calcium stores by activating IP_3_ receptors, thereby increasing intracellular Ca^2+^ concentration; QA mobilizes Ca^2+^ release from ER calcium stores, and the released Ca^2+^ can either directly promote INS exocytosis or be taken up by mitochondria to cooperatively activate oxidative metabolism and ATP synthesis, thereby enhancing GSIS. Abbreviations: *C. ficifolia* extract: fruit extract of *Cucurbita ficifolia* Bouché, and QA: Quinic acid. All images were produced using Figdraw.

In addition to the KATP–VDCC pathway described above, certain natural products can also trigger insulin secretion by activating inositol 1,4,5-trisphosphate (IP_3_) receptors on the endoplasmic reticulum (ER) membrane, thereby mobilizing intracellular calcium stores to release Ca^2+^. IP_3_ is an important intracellular second messenger generated by phospholipase C (PLC)-mediated hydrolysis of phosphatidylinositol 4,5-bisphosphate (PIP_2_) in the plasma membrane. As a soluble signaling molecule, IP_3_ diffuses within the cytoplasm and binds to IP_3_ receptors on the ER membrane. The IP_3_ receptor itself is a calcium channel, and its activation induces the release of Ca^2+^ from ER calcium stores, thereby initiating the calcium-dependent insulin exocytotic process. Studies have shown that various natural products can promote insulin secretion by modulating this pathway (Figure 4). For example, *Cucurbita ficifolia* Bouché, which possesses glucose-lowering, antioxidant, and anti-inflammatory properties [29], has a fruit extract (*C. ficifolia* extract) that can activate IP_3_ receptors on the ER, prompting the release of Ca^2+^ from calcium stores and thereby increasing intracellular calcium concentration in RINm5F cells [30]. Similarly, quinic acid (QA), widely present in various medicinal plants as well as in plants such as coffee, bilberry, and prune [31,32,33], can also mobilize Ca^2+^ release from ER calcium stores; the released Ca^2+^ is subsequently taken up by mitochondria to cooperatively activate oxidative metabolism and ATP synthesis, ultimately enhancing GSIS in INS-1E cells and isolated mouse islets [34].

#### 3.1.3. Indirect Enhancement of Insulin Secretion Through Modulation of the Incretin System

**Figure 5:**
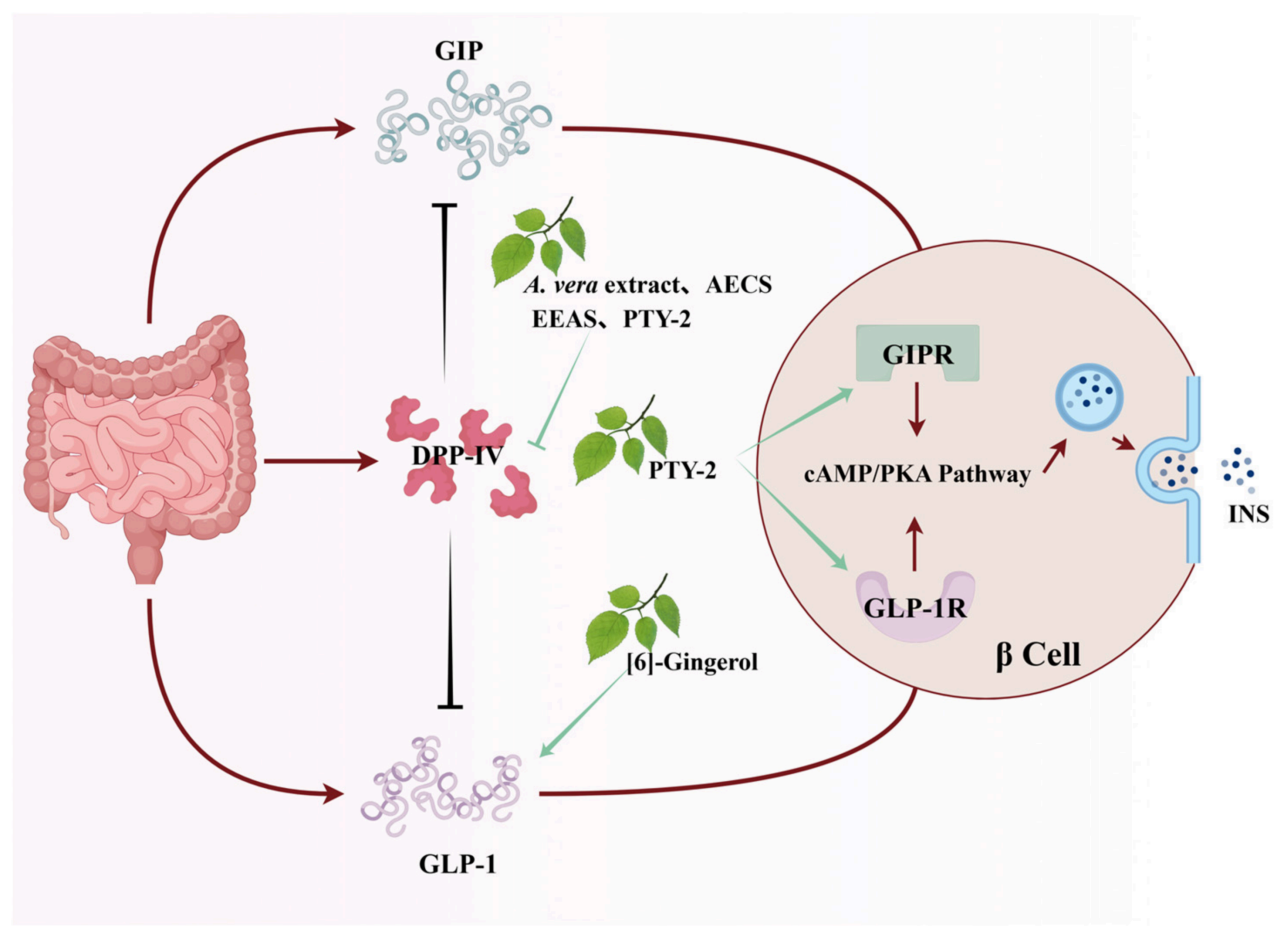
Natural products promote insulin secretion by modulating the incretin system. GLP-1 and GIP promote insulin secretion in a glucose-dependent manner. Natural products can enhance this signaling through two mechanisms: one is inhibiting DPP–IV to delay the degradation of endogenous GLP-1 and GIP, and the other is direct agonism at GLP-1R or GIPR, or an elevation of GLP-1 levels, thereby activating the cAMP/PKA pathway. *A. vera* extract, AECS, EEAS, and PTY-2 can all inhibit DPP–IV to slow the degradation of GIP and GLP-1; in addition, PTY-2 can also act as a direct agonist at GLP-1R and GIPR, while [6]-Gingerol elevates GLP-1 levels, thereby activating the cAMP/PKA pathway and promoting INS exocytosis. Abbreviations: *A. vera* extract: extract of *Aloe vera* (L.) Burm.f.; AECS: Aqueous extract of *Camellia sinensis*; EEAS: ethanolic extract of *Annona squamosa*; PTY-2: tuber aqueous extract of *Pueraria tuberosa*. All images were produced using Figdraw.

GLP-1 and glucose-dependent insulinotropic polypeptide (GIP) are the two major incretins, both of which promote insulin secretion in a glucose-dependent manner. Inhibiting their degrading enzyme DPP–IV prolongs the duration of action of endogenous incretins, whereas direct agonism at GLP-1R or GIPR activates the downstream cAMP/PKA signaling pathway. Studies have demonstrated that a variety of natural products can modulate this system (Figure 5). For example, the extract of *Aloe vera* (L.) Burm.f. (*A. vera* extract), which possesses anti-T2DM activity [35], can delay the degradation of endogenous GLP-1 by inhibiting DPP–IV activity, thereby promoting insulin secretion; concurrently, this extract can also improve lipid metabolism, alleviate lipotoxicity, and enhance insulin sensitivity in obese rats [36,37]. The aqueous extract of *Camellia sinensis* (AECS), previously described to have direct insulinotropic effects in BRIN–BD11 cells and isolated mouse islets, has also been confirmed to possess DPP–IV inhibitory activity in vitro [21,38]. The ethanolic extract (EEAS) of *Annona squamosa* [39], distributed in South Asia, contains three components: rutin, proanthocyanidin, and squafosacin G. This extract not only directly stimulates insulin secretion in a KATP channel-independent and partially cAMP-dependent manner in BRIN–BD11 cells and isolated mouse islets, but also attenuates GLP-1 degradation through DPP–IV inhibition and improves glucose tolerance and GLP-1 (7–36) levels in high-fat-fed rats [40]. Similarly, *Pueraria tuberosa*, which is rich in various active ingredients, has a tuber aqueous extract PTY-2 that exerts antidiabetic effects via a dual mechanism: on the one hand, it inhibits DPP–IV to elevate plasma GLP-1 and GIP levels; on the other hand, its active components can directly act as agonists of GLP-1R and GIPR, conferring protective effects through upregulating B-cell lymphoma 2 (Bcl-2) expression, inhibiting β-cell apoptosis, promoting insulin secretion, and restoring islet architecture in diabetic rodent models [41]. [6]-Gingerol, one of the major active constituents of *Zingiber officinale* Roscoe, exerts protective effects on pancreatic β cells in close association with the activation of the GLP-1 signaling pathway. This compound can increase GLP-1 levels, thereby enhancing the activity of the downstream cAMP/PKA/CREB signaling cascade and ultimately promoting the exocytosis of insulin granules in type 2 diabetic mice and isolated mouse islets [42].

#### 3.1.4. Other Direct Insulinotropic Mechanisms

Certain natural products can directly promote insulin secretion from β cells through unique signaling mechanisms (Table 2). For example, *Costus igneus*, used in the treatment of diabetes, has a leaf extract (Ci leaves extract) that upregulates glucokinase activity in β cells, downregulates glucose-6-phosphatase (G-6-pase) activity, and significantly enhances the expression of insulin and glucose transporter 2 (GLUT2) genes in human hematopoietic stem cell-derived β-like cells, thereby improving cellular glucose sensing and metabolic capacity [43]. Furthermore, *Nigella sativa*, widely applied in the fields of food and medicine, contains the active constituent thymoquinone, which alters the intracellular redox state by consuming nicotinamide adenine dinucleotide phosphate (NAD(P)H) and generating H_2_O_2_, thereby increasing the glucose-dependent ATP/ADP ratio and directly enhancing insulin secretion in INS-1 832/13 cells and isolated mouse islets [44]. *Commiphora myrrha* [45], which possesses antidiabetic activity, can rapidly promote insulin secretion in MIN6 cells, isolated mouse islets, and isolated human islets, and its mechanism involves the direct activation or modification of signaling pathways associated with stimulus-secretion coupling in β cells. The genus *Scoparia*, widely distributed in tropical and subtropical regions, contains the active constituent coixol, which effectively promotes insulin secretion in isolated mouse islets and MIN6 cells, and this effect is more pronounced under high-glucose conditions [46]. Steviophethanoside, isolated from *Stevia rebaudiana* Bertoni, can also enhance insulin secretion in rat INS-1 cells in a dose-dependent manner under high-glucose conditions [47].

### 3.2. Preservation of Pancreatic β-Cell Function and Mass and Improvement of Islet Homeostasis

The progression of T2DM is closely associated with the progressive decline of pancreatic β-cell function and an absolute or relative reduction in β-cell mass. Therefore, protecting β cells, inhibiting their apoptosis, and improving overall islet homeostasis represent an important intervention direction for delaying T2DM progression and preserving β-cell function. Natural products can protect pancreatic β cells through multifaceted, multi-target mechanisms of action, specifically encompassing the following aspects: (1) Maintenance of the differentiated phenotype of pancreatic β cells and promotion of regeneration, which stimulate the self-renewal and repair potential of β cells by regulating core transcription factors such as pancreatic duodenal homeobox factor-1 (Pdx-1), the Notch signaling pathway, and epigenetic modifications; (2) alleviation of oxidative stress, which protects β cells from oxidative damage by enhancing the endogenous antioxidant defense system, activating the nuclear factor erythroid 2-related factor 2 (Nrf2) pathway, or directly scavenging reactive oxygen species (ROS); (3) inhibition of apoptosis and endoplasmic reticulum stress induced by toxic aggregation of hIAPP, involving regulation of the balance of apoptosis-related proteins such as Bcl-2/BCL-2-associated X protein (Bax), suppression of the caspase cascade and NOD-like receptor thermal protein domain associated protein 3 (NLRP3) inflammasome activation, as well as blocking abnormal hIAPP aggregation and its downstream toxic effects; and (4) attenuation of local islet inflammatory responses, which improves the microenvironment for β-cell survival by inhibiting key pro-inflammatory signaling pathways such as nuclear factor kappa-light-chain-enhancer of activated B cells (NF-κB). This section will elaborate on each of these protective mechanisms in detail following this framework.

#### 3.2.1. Maintenance of the Differentiated Phenotype of Pancreatic β Cells and Promotion of Regeneration

Maintaining the differentiated phenotype of pancreatic β cells and promoting their regeneration represent important strategies for restoring insulin secretory capacity and delaying the progression of T2DM. Existing evidence indicates that a variety of natural active ingredients can effectively induce β-cell proliferation and improve their function, with mechanisms involving the following multiple regulatory layers.

**Figure 6:**
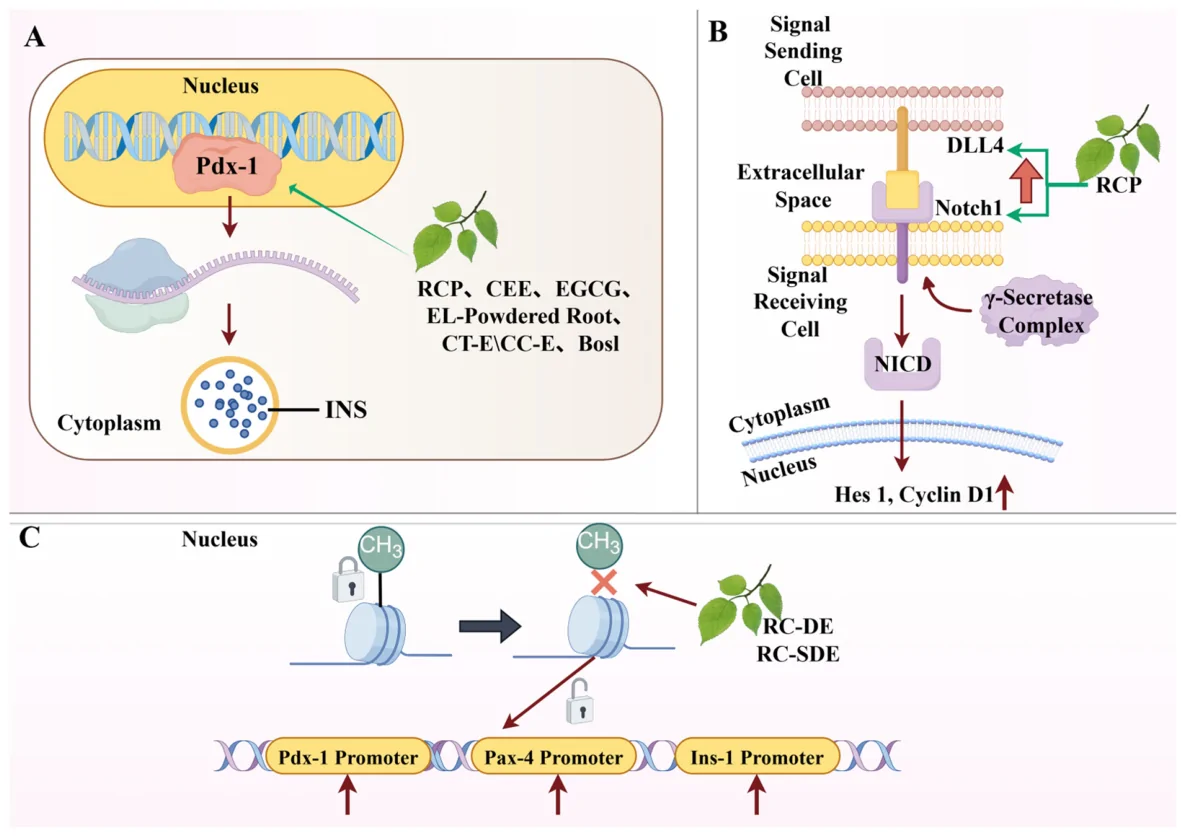
Natural products maintain the differentiated phenotype and promote the regeneration of pancreatic β cells by regulating Pdx-1, Notch signaling, and demethylation. (**A**) Multiple natural products improve β-cell function by upregulating Pdx-1 expression or activity: RCP, CEE, EGCG, EL-Powdered Root, CT-E/CC-E, and Bosl upregulate Pdx-1 expression in β cells, thereby increasing INS production. (**B**) The Notch signaling pathway can maintain cells’ proliferative potential and undifferentiated state, inhibit premature differentiation, and provide a regulatory basis for β-cell regeneration. RCP can upregulate the expression of Notch1 and its ligand DLL4, promoting the transcription of downstream Hes1 and Cyclin D1. (**C**) Natural products can also indirectly affect the transcriptional activity of key genes such as *Pdx-1*, *Pax-4*, and *Ins-1* by intervening in epigenetic modifications. RC-DE and RC-SDE can reduce the methylation levels in the promoter regions of *Pdx-1*, *Pax-4*, and *Ins-1* genes, thereby regulating the expression of genes associated with pancreatic β-cell function and regeneration. Abbreviations: RCP: pectin-like polysaccharide from *Rosa canina*; CEE: methanolic extract of *Centaurium erythraea* Rafn; EGCG: Epigallocatechin-3-gallate; EL-Powdered Root: processed root powder of Eurycoma longifolia Jack; CT-E: *Cinnamomum tamala* extract; CC-E: *Cinnamomum cassia* extract; Bosl: Boschnaloside; RC-DE: decoction extract of Rosa canina; RC-SDE: spray-dried extract of *Rosa canina*. All images were produced using Figdraw.

Pdx-1, as a core transcription factor governing pancreatic organogenesis and the maintenance of mature β-cell function, plays a pivotal role in downstream transcriptional regulatory networks. Pdx-1 can directly bind to the insulin gene promoter region, serving as a key molecular switch that drives insulin biosynthesis and glucose-stimulated insulin secretion; it is also one of the important factors maintaining the differentiated state of mature β cells [75]. Currently, a variety of natural products have been demonstrated to improve β-cell function by increasing the expression or activity of Pdx-1. For example, *Rosa canina* possesses multiple biological activities, including antioxidant and anti-inflammatory effects [76,77], and the pectin-like polysaccharide isolated from *Rosa canina* (RCP) can improve β-cell function by increasing Pdx-1 expression in RIN-5F cells and streptozotocin (STZ)-induced diabetic rats [78,79]. *Centaurium erythraea* Rafn (CE) is traditionally used as a food and herbal medicine [80,81] and possesses hypoglycemic, antipyretic, carminative, and detoxifying effects [82,83]; its methanolic extract (CEE) can improve insulin secretion by modulating the transcript levels of Pdx-1 and MafA in Rin-5F cells and Wistar rats [84]. *Eurycoma longifolia* Jack (EL) has multiple pharmacological activities including immunomodulation, stress relief, and antitumor effects [85,86,87], and its processed root powder (EL-Powdered Root) can induce Pdx-1 expression, thereby promoting β-cell proliferation and neogenesis in C57BL/6J and db/db mice (BKS.Cg-Dock7m^+/+^ Lepr^db^/JNarl) [88]. Cinnamon is rich in various active constituents such as cinnamaldehyde, eugenol, and polyphenolic compounds, and exerts positive regulatory effects on glucose metabolism, insulin sensitivity, and pancreatic β-cell function [89,90]; extracts obtained from *Cinnamomum tamala* and *Cinnamomum cassia* (*Cinnamomum tamala* extract, CT-E, and *Cinnamomum cassia* extract, CC-E) can partially restore the impaired Pdx-1 transcript levels under lipotoxic conditions and thereby improve insulin secretion in INS-1 cells, MIN6 cells, and isolated murine islets [91]. Boschnaloside (Bosl), one of the major active constituents of the traditional Chinese herbal medicine *Boschniakia rossica*, can increase intracellular insulin protein content and upregulate Pdx-1 expression in β cells under glucotoxic conditions in BRIN–BD11 cells under glucotoxic conditions [92]. Epigallocatechin-3-gallate (EGCG), one of the major active constituents of green tea [93], can upregulate Pdx-1 expression in β cells of C57BL/6 mice, thereby increasing insulin secretion [94,95]. These studies collectively reveal that enhancing the expression or activity of Pdx-1 is a key shared mechanism through which numerous structurally diverse natural products exert β-cell protective and function-promoting effects (Figure 6A).

The Notch signaling pathway provides a necessary regulatory basis for β-cell regeneration by maintaining the proliferative potential and undifferentiated state of cells and inhibiting premature directed differentiation. This pathway is widely present in vertebrates and invertebrates and participates in the regulation of multiple morphogenetic processes such as cell differentiation, apoptosis, and proliferation. In islets, the activation of Notch signaling generally inhibits the premature differentiation of progenitor cells and maintains their undifferentiated state, and it also serves as an important target for the actions of natural products. Studies have shown that the pectin-like polysaccharide from *Rosa canina* (RCP) can upregulate the expression of Notch1 and Delta-like ligand 4 (DLL4), promoting the transcription of downstream Hes Family BHLH Transcription Factor 1 (Hes1) and Cyclin D1, thereby driving β-cell proliferation in RIN-5F cells and STZ-induced diabetic rats [78,79] (Figure 6B).

In addition to directly regulating transcription factors, natural products can also indirectly influence the transcriptional activity of key genes such as *Pdx-1* through intervening in epigenetic modifications. Epigenetic modifications primarily alter chromatin structure through chemical modifications such as DNA methylation and histone modification, thereby regulating gene expression; notably, methylation of the DNA promoter region typically leads to transcriptional repression. Studies have demonstrated that the decoction extract of *Rosa canina* (RC-DE) and its spray-dried powder (RC-SDE) can reduce the methylation levels of the promoter regions of *Pdx-1*, *Pax-4*, and *Ins-1* genes, thereby regulating the expression of genes associated with pancreatic β-cell function and regeneration in Wistar rats [77] (Figure 6C).

Beyond the above mechanisms, certain natural products can also stimulate β-cell regen Beyond the above mechanisms, certain natural products can also stimulate β-cell regeneration or improve their function through other pathways. For example, the traditional herbal medicine *Cichorium intybus* L. is widely used in folk medicine for the treatment of various diseases [96,97]; its leaf extract (*C. intybus* leaf extract, CLE) can effectively induce the differentiation of P19 embryonal carcinoma cell line into insulin-secreting cells in vitro [49]. *Curcuma amada* possesses multiple biological activities, including antioxidant, anti-inflammatory, and antidiabetic effects [98,99,100], and the ethyl acetate fraction of its rhizome extract (EtOAc fraction of *C. amada* rhizome extract, CA-RE) can stimulate β-cell regeneration and increase insulin secretion in Wistar rats [50]. *Emblica officinalis*, widely distributed in tropical and subtropical regions such as China and Pakistan, has antidiabetic effects from its fruit [101,102,103]; its major constituent, ellagic acid (EA), can stimulate glucose-induced insulin secretion and improve β-cell morphology and number in STZ-induced diabetic rats and isolated mouse islets [51]. *Hibiscus rosa-sinensis*, widely cultivated in tropical regions, has multiple medicinal values, including antitumor, antihypertensive, and antioxidant activities [104,105], and its flower extract (hydroalcoholic extract of flower *Hibiscus rosa-sinensis*, HEFHR) can promote pancreatic β-cell regeneration and improve β-cell morphology and number in alloxan-induced Wistar rats [52] (Table 2).

#### 3.2.2. Protection of Pancreatic β-Cell Viability and Improvement of Function Through Alleviation of Oxidative Stress

Oxidative stress is a key pathological factor leading to pancreatic β-cell damage and dysfunction. Due to the relatively low expression of endogenous antioxidant enzyme systems, β cells are exceptionally sensitive to ROS. Under metabolic stress conditions such as hyperglycemia and hyperlipidemia, mitochondrial dysfunction, endoplasmic reticulum stress, and their downstream activated signaling pathways can promote excessive ROS generation, which in turn causes DNA damage, inactivation of protein function, and lipid peroxidation, ultimately resulting in apoptosis and impaired insulin synthesis and secretion. Therefore, suppressing oxidative stress is one of the important strategies for protecting β-cell function and survival.

The intracellular endogenous antioxidant defense system mainly includes superoxide dismutase (SOD), glutathione peroxidase (GPx), catalase (CAT), and reduced glutathione (GSH), which act synergistically to eliminate ROS. The content of malondialdehyde (MDA), the end product of lipid peroxidation, is a key indicator for evaluating the degree of oxidative damage. Correspondingly, elevating antioxidant enzyme activities or GSH levels and reducing MDA content are regarded as direct experimental evidence of attenuated oxidative stress. A large body of research has demonstrated that natural active ingredients, represented by phenolics and flavonoids, can effectively alleviate oxidative stress-induced β-cell damage through multiple mechanisms, including enhancing the endogenous antioxidant enzyme system, activating the Nrf2 pathway, and directly scavenging free radicals.

**Figure 7:**
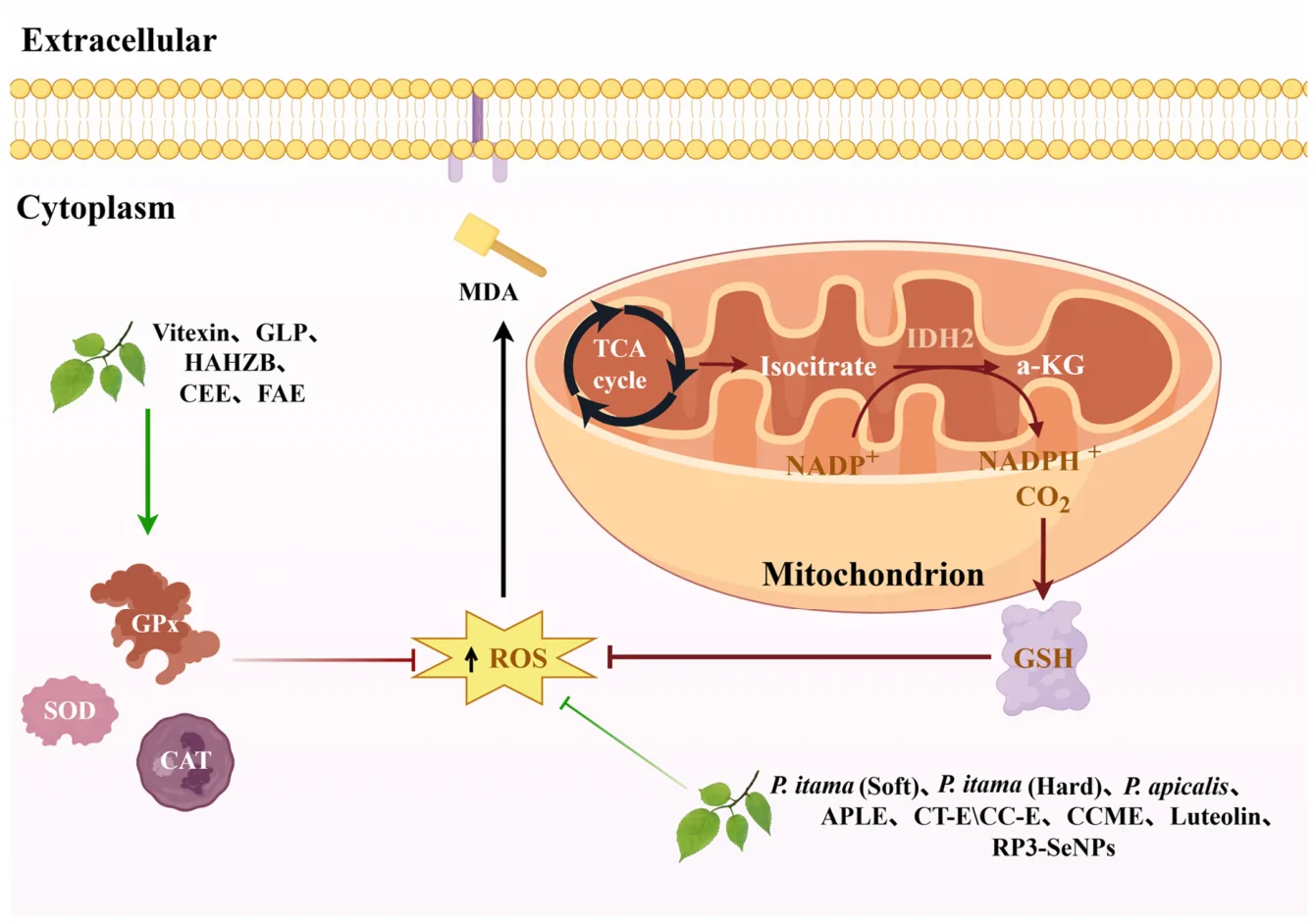
Natural products protect β cells by enhancing antioxidant capacity and directly scavenging free radicals. Oxidative stress is a key driver of pancreatic β-cell damage. Natural products attenuate oxidative damage by enhancing the endogenous antioxidant defense system, as exemplified by Vitexin, GLP, HAHZB, CEE, and FAE, or by directly suppressing excessive ROS production. *P. itama* (Soft), *P. itama* (Hard), *P. apicalis*, APLE, CC-E, CT-E, CCME, Luteolin, and RP3-SeNPs can directly inhibit the excessive production of intracellular ROS. Abbreviations: GSH: Glutathione; IDH2: Isocitrate Dehydrogenase (NADP(+)) 2; α-KG: α-Ketoglutaric Acid; NADP^+^: Nicotinamide Adenine Dinucleotide Phosphate; NADPH: reduced Nicotinamide Adenine Dinucleotide Phosphate; GLP: polysaccharide fraction extracted from *G. lithophila*; HAHZB: active component extracted from the bark of *Homalium zeylanicum* Benth.; CEE: methanolic extract of *Centaurium erythraea* Rafn; FAE: ethanolic extract of *Ferula assa-foetida* L.; APLE: *Abrus precatorius* leaf extract; CC-E: *Cinnamomum cassia* extract; CT-E: *Cinnamomum tamala* extract; CCME: methanolic extract of *Crassocephalum crepidioides.* All images were produced using Figdraw.

Enhancing the endogenous antioxidant system is one of the primary mechanisms by which natural products exert protective effects (Figure 7). For example, vitexin, isolated from *Ficus deltoidea* [106,107], which has antidiabetic potential, can enhance antioxidant capacity by increasing GPx activity and reducing thiobarbituric acid reactive substances (TBARS) levels, thereby promoting β-cell regeneration and improving pancreatic architecture in STZ-induced diabetic rats [108]. The crude polysaccharide and rhamnose-enriched polysaccharide derived from *G. lithophila* (GLP) can increase SOD and GPx activities and reduce MDA content in the liver, kidney, and pancreatic tissues of STZ-induced diabetic Wistar rats, thus alleviating oxidative damage and inflammatory responses [109]. The bark of *Homalium zeylanicum* Benth. possesses various activities including antioxidant, antibacterial, hypolipidemic, anthelmintic, anti-inflammatory, hypoglycemic, and hepatoprotective effects [110]; the bioactive fraction HAHZB isolated from it can effectively mitigate oxidative stress by increasing pancreatic SOD, CAT, and GSH levels and reducing MDA content in Wistar rats [111]. The methanolic extract of *Centaurium erythraea* Rafn (CEE), previously mentioned, can also alleviate STZ-induced oxidative stress in Rin-5F cells and Wistar rats, as evidenced by reduced DNA damage, lipid peroxidation, and protein S-glutathionylation, normalized antioxidant enzyme activities, and modulation of the activity of oxidative stress-sensitive transcription factors such as NF-κB-p65, Nrf2, and Specificity Protein 1 (Sp1), thereby activating pro-survival signaling pathways such as Akt and ERK to promote β-cell survival and proliferation [84]. Furthermore, the ethanolic extract of the oleo gum resin of *Ferula assa-foetida* L. (FAE), a traditional medicinal plant distributed in Iran, Afghanistan, and parts of India, can elevate pancreatic antioxidant enzyme activities, stimulate β-cell proliferation and regeneration, and restore islet structure and function in STZ-induced diabetic Wistar rats [112].

Phenolic and flavonoid compounds, owing to their chemical structures rich in phenolic hydroxyl groups, can donate electrons or hydrogen atoms to directly neutralize free radicals and interrupt radical chain reactions, thereby exerting direct antioxidant effects. As common dietary bioactive ingredients, the antioxidant, anti-inflammatory, and antidiabetic properties of polyphenolic compounds have been extensively studied [113,114,115]. For example, different types of propolis (*P. itama* (Soft), *P. itama* (Hard), *P. apicalis*) can promote pancreatic β-cell regeneration through their antioxidant and anti-inflammatory actions in male Sprague–Dawley rats [116]. The leaf extract of *Abrus precatorius* (APLE) [117], which has therapeutic potential for T2DM, is rich in phenolic and flavonoid constituents, can directly scavenge free radicals and possesses ferric reducing ability, and these effects synergistically alleviate oxidative stress, thereby restoring the number and function of pancreatic β-cells in alloxan-induced diabetic Sprague–Dawley rats [118]. The extracts CT-E and CC-E from *C. cassia* and *C. tamala*, previously mentioned, in addition to regulating Pdx-1 expression, can also attenuate palmitic acid (PA)-induced β-cell apoptosis and inhibit hydrogen peroxide-stimulated ROS production, thus mitigating oxidative stress and lipotoxicity in INS-1 cells, MIN6 cells, and isolated murine islets [91]. The protective effects of some natural products can extend to the regulation of organelle function and apoptosis signaling pathways. For instance, the complex RP3-SeNPs, constructed from the polysaccharide RTFP-3 isolated from the fruit of *Rosa roxburghii* Tratt [119], which possesses multiple functions including antioxidant, antitumor, and antidiabetic activities, can effectively block excessive intracellular ROS production, prevent the loss of mitochondrial membrane potential, inhibit the activation of Caspase-3, -8, and -9, and improve GSIS function by downregulating uncoupling protein-2 (UCP-2) expression in INS-1 cells [120]. In addition, the methanolic extract of *Crassocephalum crepidioides* (CCME), widely distributed in tropical and subtropical regions, exhibits significant antioxidant activity, can scavenge excess intracellular ROS, and reduce the apoptosis rate of β cells in INS-1 cells and Wistar rats [121]. Finally, luteolin not only possesses antioxidant [122] and insulin secretion-promoting activities [123], but can also effectively scavenge ROS and improve palmitic acid-induced β-cell apoptosis and GSIS dysfunction by promoting autophagic flux in INS-1E cells, isolated mouse islets, and STZ-induced diabetic C57 mice [124] (Figure 7).

Moreover, the protective effect of *Uncaria gambir* on pancreatic β-cells may be related to the antioxidant and pro-regenerative capacities of its rich catechins and quercetin in STZ-induced diabetic male Sprague–Dawley rats [125]. The ethanolic extract ST3 of *Stevia rebaudiana* Bertoni can alleviate endoplasmic reticulum stress by upregulating chaperone proteins and downregulating ER stress markers, thereby improving mitochondrial function in INS-1E cells and human pancreatic islets [126] (Table 2).

Activation of the core antioxidant transcription factor Nrf2 is another key protective pathway. Nrf2, as the central regulator of the cellular oxidative stress response, is responsible for initiating the expression of a variety of endogenous antioxidant enzymes and plays a critical role in maintaining cellular redox homeostasis. Studies have shown that silibinin, extracted from *Silybum marianum*, in addition to possessing multiple pharmacological activities such as antioxidant, hepatoprotective, and antidiabetic effects [127], can activate the Nrf2 pathway and specifically upregulate the expression of downstream effector molecules heme oxygenase-1 (HO-1) and SOD2, thereby effectively reducing excessive ROS generation induced by high-glucose and high-lipid conditions, significantly improving β-cell survival, and promoting insulin secretion in INS-1 and NIT-1 cells and in high-fat diet/STZ-induced diabetic Sprague–Dawley rats [53] (Table 2).

In addition, various other natural products also exert β-cell protective effects by enhancing antioxidant capacity. The whole seeds and cold-pressed seed oil of *Nigella sativa* (NSSO) are used in the treatment of multiple diseases, and both can protect pancreatic tissue through antioxidant effects, reduce β-cell apoptosis, and partially promote their regeneration, showing potential for the treatment of T2DM in STZ-induced diabetic Sprague–Dawley rats [54,55,56,128]. The extract of *Polysiphonia japonica* (PJE), which possesses antioxidant activity, can alleviate PA-induced oxidative stress and improve β-cell function by inhibiting the bone morphogenetic protein (BMP) signaling pathway in Ins-1 cells, isolated mouse islets, and transgenic zebrafish embryos [57]. A series of traditional medicinal plants and their active ingredients, such as the aqueous extract of *Stevia rebaudiana* Bertoni (SRE) in alloxan-induced NMRI mice [58], Ginseng oligopeptides (GOPs) in high-carbohydrate/high-fat diet and alloxan-induced SD rats [59], madecassoside from *Centella asiatica* (L.) Urb. in STZ/nicotinamide-induced SD rats [60], *Leea macrophylla* root extract (LMR) in fructose-fed STZ-induced Wistar rats [61], seed oil of *Citrullus lanatus* (SMSO) in high-fat/sugar diet and STZ-induced SD rats [16], the ethanolic extract of *Quercus liaotungensis* Koidz (QLKAE) in high-sugar/fat diet and STZ-induced SD rats [62], and betulin from birch bark in nicotinamide/STZ-induced Wistar rats [63], have all been reported to enhance the antioxidant defense capacity of pancreatic β-cells through various mechanisms and effectively alleviate oxidative stress damage (Table 2).

In summary, through intricate mechanisms such as enhancing the endogenous antioxidant enzyme system, activating the Nrf2 pathway, directly scavenging free radicals, protecting organelle function, and influencing multiple signaling pathways, natural products attenuate oxidative stress-induced damage to β-cells at different levels, providing a rich material basis and theoretical foundation for improving their function and survival.

#### 3.2.3. Inhibition of β-Cell Apoptosis and Pyroptosis

Apoptosis is a key pathological process leading to the progressive loss of pancreatic β-cell mass. As a form of programmed cell death regulated by genes, apoptosis is essential for maintaining tissue homeostasis under physiological conditions; however, its aberrant activation is a critical contributor to β-cell failure in diabetes. Therefore, inhibiting β-cell apoptosis is an important strategy for preserving their population size and function. Studies have demonstrated that a variety of natural products can effectively prevent the programmed death of β cells by modulating the expression of apoptosis-related proteins, suppressing apoptotic signaling pathways, or preventing the aggregation of toxic species.

The core execution machinery of apoptosis primarily involves the Bcl-2 family of proteins and the caspase protease cascade. The Bcl-2 family includes anti-apoptotic members (e.g., Bcl-2, Bcl-xl) and pro-apoptotic members (e.g., Bax). The dynamic balance between them determines the permeability of the mitochondrial outer membrane. When the Bax/Bcl-2 ratio decreases, it favors the maintenance of mitochondrial membrane integrity, prevents the release of cytochrome c into the cytoplasm, and thereby inhibits the activation of downstream initiator caspase-9 and effector caspase-3, ultimately blocking the execution of the apoptotic program.

**Figure 8:**
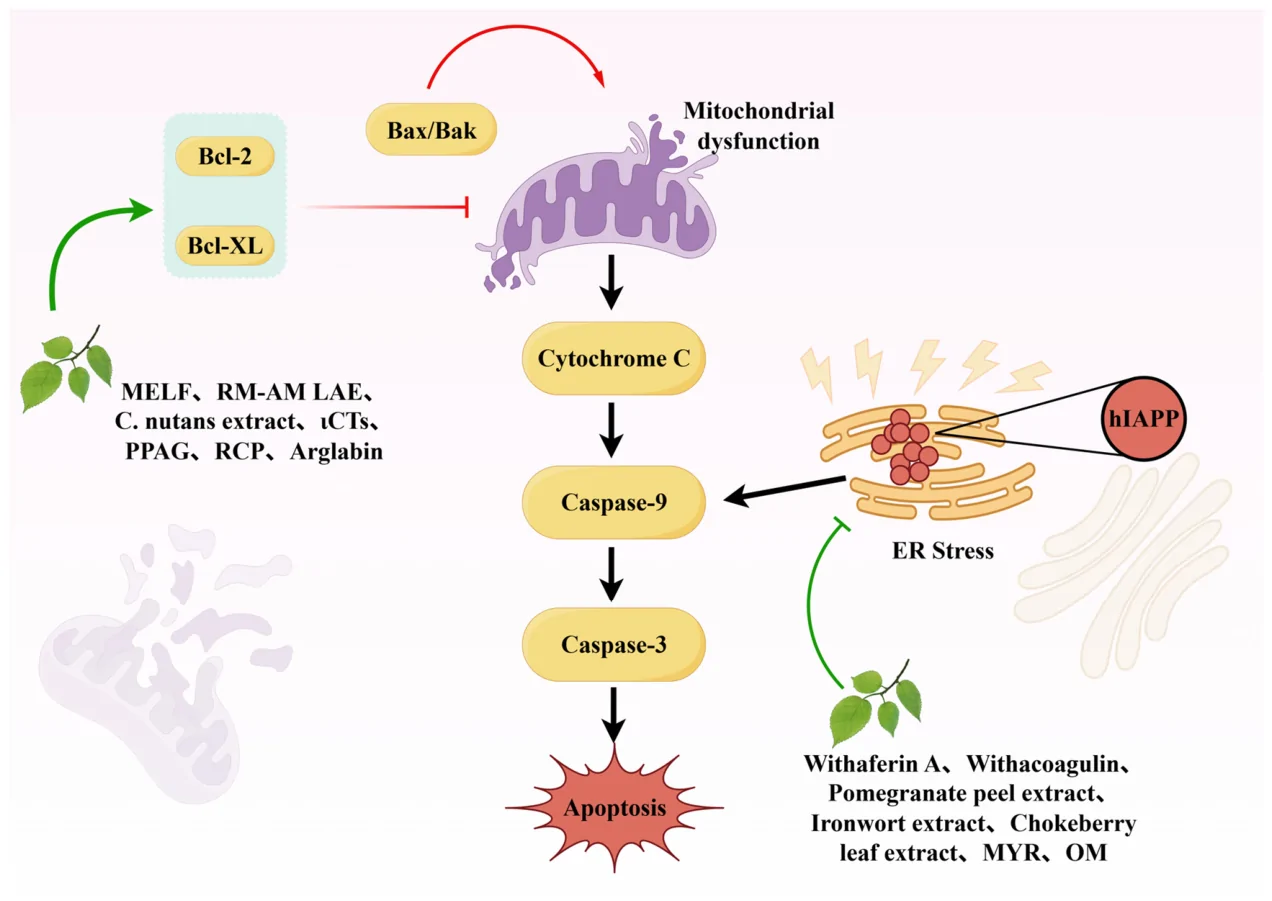
Natural products protect pancreatic β cells by inhibiting apoptotic signaling and toxic hIAPP aggregation. Natural products inhibit β-cell apoptosis through two main pathways. (1) The regulation of apoptosis-related proteins—MELF, RM-AM LAE, *C. nutans* extract, ιCTs, PPAG, RCP, and Arglabin—reduces β-cell apoptosis by modulating the Bax/Bcl-2 ratio. (2) The inhibition of toxic hIAPP aggregation—Withaferin A, Withacoagulin, Pomegranate peel extract, Ironwort extract, chokeberry leaf extract, MYR, and OM—inhibits hIAPP aggregation, thereby preventing β-cell destruction by toxic aggregates. Abbreviations: ER: Endoplasmic reticulum; MELF: methanolic extract of *L. flavescens*; RM-AM LAE: leaf aqueous extract of *Rhizophora mucronata* and *Avicennia marina*; *C. nutans* extract: ethanolic extract of leaves of *Clinacanthus nutans*; ιCTs: ι-carrageenan tetrasaccharide; PPAG: phenylpropenoic acid glucoside from *Aspalathus linearis*; RCP: pectin-like polysaccharide from *Rosa canina*; MYR: Myricetin; OM: Oligomannuronate. All images were produced using Figdraw.

Numerous natural products have been demonstrated to act on this critical regulatory node (Figure 8). For example, the methanolic extract of the traditional herb *L. flavescens* (MELF) can upregulate the expression of anti-apoptotic proteins Bcl-2 and Bcl-xl while downregulating the levels of cleaved caspase-9 and cleaved caspase-3, thereby inhibiting the mitochondrial pathway of apoptosis in INS-1 cells and nicotinamide/STZ-induced diabetic Sprague–Dawley rats [129]. The leaf aqueous extract of *Rhizophora mucronata* and *Avicennia marina* (RM-AM LAE) can significantly reduce the proportion of caspase-3-positive β cells in islets of STZ-induced diabetic Wistar rats, effectively suppressing β-cell apoptosis [130]. *Clinacanthus nutans* [131,132], which has antidiabetic potential, has an ethanolic leaf extract (*C. nutans* extract) that inhibits c-Jun N-terminal kinase (JNK) activation through its dual antioxidant and anti-inflammatory activities, consequently reducing caspase-3 expression in STZ-induced diabetic Wistar rats [72]. ι-Carrageenan, widely used as a pharmaceutical excipient and additive in drug and food processing [133], also possesses various activities including antiviral, antioxidant, and antitumor effects [134]; the novel oligosaccharide ι-carrageenan tetrasaccharide (ιCTs) prepared from ι-carrageenan using marine enzyme Cgi82A can activate the GLP-1/cAMP/PKA signaling pathway, downregulate pro-apoptotic proteins, and upregulate Bcl-2, thereby inhibiting the mitochondrial apoptotic pathway in high-fat/high-sucrose diet-fed C57BL/6J mice [135]. Phenylpropenoic acid glucoside (PPAG), derived from *Aspalathus linearis*, has a hypoglycemic effect [136] and can counteract β-cell apoptosis by maintaining Bcl-2 expression and inhibiting caspase activation in STZ-treated Balb/c mice, INS-1E cells, and human islets [137]. The previously mentioned RCP from *Rosa canina*, while promoting β-cell proliferation, has also been shown to inhibit apoptosis by reducing the Bax/Bcl-2 ratio in RIN-5F cells and STZ-induced diabetic rats [78,79]. Arglabin, extracted from *Artemisia glabella*, can inhibit NLRP3 inflammasome activation, block caspase-1 maturation and Interleukin-1β (IL-1β) conversion, while modulating the Bcl-2/Bax ratio and inducing autophagy in high-fat diet-fed ApoE2.Ki mice [138].

Human islet amyloid polypeptide (hIAPP) is a 37-amino acid peptide hormone that is co-localized, co-packaged, and co-secreted with insulin in β cells. Aberrant aggregation of hIAPP and the resulting endoplasmic reticulum stress represent another important factor leading to β-cell apoptosis, and a variety of natural products can exert protective effects by inhibiting hIAPP aggregation (Figure 8). Withaferin A and withacoagulin from *Ashwagandha* can inhibit hIAPP aggregation, thereby preventing damage to β cells from toxic aggregates in silico [139]. Pomegranate peel extract, ironwort extract, and chokeberry leaf extract can directly inhibit the conformational transition of hIAPP to β-sheet structures, prevent the formation of toxic oligomers and fibrils, and reduce hIAPP-induced accumulation of reactive oxygen species and mitochondrial stress in INS 832/13 cells [140]. The polyphenolic flavonoid myricetin (MYR) [141], which possesses various pharmacological effects including anti-inflammatory, antioxidant, antihypertensive, anticancer, and antidiabetic activities [142,143,144], can also effectively inhibit hIAPP aggregation and alleviate the resulting oxidative stress, lipid peroxidation, and mitochondrial membrane potential depolarization in INS-1E cells and isolated mouse islets [145]. Alginate, a polysaccharide widely used as a dietary supplement and pharmaceutical excipient with multiple bioactivities [146], has a derived oligosaccharide, oligomannuronate (OM), which can prevent hIAPP-induced mitochondrial dysfunction and apoptosis in RINm5F cells by inhibiting the activation of the JNK signaling pathway [147].

*Morus alba* leaves are among the most widely used Chinese herbal medicines [148] and can be employed in the treatment of T2DM [149]; their ethanolic extract (*Morus alba* leaves ethanol extract, MLE) can induce protective autophagy by activating the AMPK/mTOR signaling pathway in INS-1 cells and high-fat diet/STZ-induced diabetic Sprague–Dawley rats [64]. The high-molecular-weight fraction Om Santal Adivasi (OSA^®^) extracted from *Gymnema sylvestre* can directly inhibit caspase-3/7 activity triggered by inflammatory factors and regulate endoplasmic reticulum stress balance by upregulating casein kinase II, synergistically enhancing endogenous antioxidant defense to provide multi-level protection in MIN6 cells and isolated mouse islets [65] (Table 2).

In addition to the classical apoptosis pathway, NLRP3 inflammasome-mediated pyroptosis is also an important mechanism leading to β-cell death. Upon activation, the NLRP3 inflammasome cleaves caspase-1, subsequently triggering pyroptosis and promoting the maturation and release of pro-inflammatory cytokines such as IL-1β, which drives local islet inflammation and β-cell death. Inhibiting its assembly or blocking upstream signals can alleviate inflammatory damage to β cells. An epidemiological study from South Korea confirmed that smoking is an independent risk factor for the progression of diabetes, although the mechanisms by which it induces hyperglycemia had not been fully elucidated [150]. Subsequent studies have shown that cigarette smoke exposure can upregulate the expression of thioredoxin interacting protein (TXNIP), activate the NLRP3 inflammasome, and thereby induce pancreatic β-cell pyroptosis; andrographolide, however, can inhibit TXNIP upregulation and block the activation of this pathway, thus alleviating β-cell pyroptosis in C57BL/6 mice exposed to cigarette smoke and in MIN6 cells treated with cigarette smoke extract [68,151] (Table 2).

#### 3.2.4. Attenuation of Inflammatory Responses to Protect Pancreatic β Cells

In the pathological microenvironment of T2DM, chronic low-grade inflammation is one of the key factors driving pancreatic β-cell dysfunction and mass loss. Chronic hyperglycemia and hyperlipidemia can abnormally activate resident immune cells within the islets, such as macrophages, prompting them to persistently release pro-inflammatory cytokines including tumor necrosis factor-α (TNF-α) and IL-1β, thereby creating and sustaining a detrimental inflammatory microenvironment locally. These inflammatory mediators can act directly on β cells and activate key pro-inflammatory signaling pathways within them, such as NF-κB. Therefore, suppressing the excessive activation of such core pro-inflammatory signaling pathways and reducing the production of downstream pro-inflammatory mediators represent important strategies for mitigating inflammatory attacks on β cells and preserving their structure and function. Existing evidence indicates that a variety of natural products can precisely intervene in the above pathological processes.

Numerous studies have demonstrated that the anti-inflammatory protective effects of natural products are primarily achieved through targeted inhibition of the activity of key inflammatory signaling nodes, such as NF-κB, p38 mitogen-activated protein kinase (p38 MAPK), and JNK (Table 2). For example, CCB, a mixture of polyphenol-rich cocoa powder and carob flour, not only enhances the antioxidant capacity of pancreatic tissue but also reduces the levels of key pro-inflammatory cytokines such as interleukin-6 (IL-6) and TNF-α by effectively inhibiting the NF-κB signaling pathway, thereby alleviating pancreatic inflammation and exerting a protective effect on β cells in Zucker diabetic fatty (ZDF) rats [69]. Maidong polysaccharide extract (MPE), extracted from the traditional Chinese medicine Maidong (*Ophiopogonis Radix*), can effectively alleviate islet inflammatory responses by inhibiting the IKK–NF-κB inflammatory signaling axis, downregulating IL-1β expression, and reducing the nuclear translocation of NF-κB p65 protein in db/db mice (BKS.Cg-Dock7^m^+/+Lepr^db^/J), high-fat diet-induced obese C57BL/6J mice, MIN6 cells, and isolated mouse islets [70]. The ethanolic extract PMI 5011 from *Artemisia dracunculus* L. attenuates IL-1β-mediated NF-κB signaling activity by inhibiting the phosphorylation of p38 MAPK, thereby downregulating the expression of a series of inflammation-related genes in 832/13 rat insulinoma cells and dispersed islet cells from C57BL/6J mice [71]. Similarly, the anti-inflammatory effect of *C. nutans* extract is also closely associated with its inhibition of the c-Jun N-terminal kinase (JNK) pathway in STZ-induced diabetic Wistar rats [72]. Collectively, these studies indicate that the targeted regulation of key inflammatory signaling nodes, including NF-κB, p38 MAPK, and JNK, represents an important molecular mechanism by which natural products alleviate islet inflammation and maintain β-cell function.

### 3.3. Mechanisms Not Yet Fully Elucidated but with Confirmed Efficacy

In addition to the natural products with relatively well-defined molecular mechanisms described above, there remains a group of natural extracts that have demonstrated definitive islet-protective or glucose-lowering efficacy in experimental settings, yet their precise molecular targets and signaling networks have not been fully resolved. These findings are also of significant suggestive value, indicating that natural products may contain as-yet-unidentified active constituents or may act through unique pathways that have not been previously explored, and thus warrant further in-depth investigation.

Existing studies have preliminarily revealed some of the effects of such products (Table 2). For example, the seed extract AMEBO of *Berberis orthobotrys* can effectively preserve islet architecture and alleviate its pathological atrophy in alloxan-induced diabetic Sprague–Dawley rats [73]. Another African traditional medicinal plant, *Balanites aegyptiaca* [152,153], has an ethanolic extract (BA Ex) that can not only directly stimulate insulin secretion but also achieve comprehensive protection of β cells by downregulating apoptosis-related signals in pancreatic tissue and upregulating the expression of insulin receptor substrate 1 (IRS-1) in STZ-induced diabetic Sprague–Dawley rats [74]. These results not only expand the known spectrum of islet-protective effects of natural products but also provide clear clues for subsequent target identification and mechanistic elucidation.

## 4. Structure–Function Summary of Monomeric Compounds

The structural diversity of natural active monomeric compounds provides abundant pharmacophore resources for the regulation of islet function (Table 3). Among them, flavonoid monomers represented by EGCG, luteolin, and myricetin [40,53,94,95,108,124,145] cover multiple processes, including the promotion of insulin secretion, alleviation of oxidative stress, and inhibition of apoptosis, suggesting, as a preliminary trend, that this class of skeletons may possess an inherent advantage of multi-target action. Terpenoid monomers, such as boschnaloside and andrographolide [60,63,66,67,68,92,151,154,155], appear to be more focused on promoting β-cell proliferation and inhibiting apoptosis and pyroptosis. Steroidal monomers, mainly represented by withaferin A and withacoagulin [139], are currently associated primarily with the inhibition of toxic hIAPP aggregation. Other types of monomers, such as quinic acid and ellagic acid [34,40,46,47], have relatively dispersed targets, covering multiple levels from calcium signaling regulation to β-cell regeneration.

Overall, although deriving a unified structure–activity relationship (SAR) rule based on structural type remains challenging, the existing evidence indicates that natural monomeric compounds possess definite activities in promoting insulin secretion and protecting β-cell survival and function. However, it is important to emphasize that the structural preferences described above should be regarded as preliminary trends rather than established SAR, since the number of structurally characterized compounds is still limited and their direct molecular targets are often unknown. These observations therefore remain hypotheses that require further validation.

Notably, natural products essentially exist in the form of complex mixtures, and the vast array of pharmacophore molecules contained therein constitutes a reservoir of active molecules that remains to be systematically explored; even for those monomeric components whose mechanisms are not yet clarified, their potential research value as unique chemical entities should not be overlooked. Some monomers have already exhibited characteristics of synergistic action through multiple mechanisms, suggesting that they may exert comprehensive islet-protective effects by simultaneously intervening in multiple pathological processes.

However, the direct molecular targets and their upstream and downstream signaling networks of most monomers remain incompletely elucidated, and SAR studies are still at an early stage. Future work may focus on addressing the key bottleneck that has long constrained the development of this field, namely the identification of targets of natural products. In recent years, cell membrane chromatography (CMC) technology, as an important methodological breakthrough in the field of affinity screening, can simulate the specific binding of drug molecules to cell membrane receptors in their native conformations, providing a powerful tool for the high-throughput and highly sensitive screening of active components targeting β-cell membrane receptors from complex extraction systems. Combining candidate components obtained through CMC screening with techniques such as chemical proteomics, molecular docking, and surface plasmon resonance (SPR) is expected to systematically pinpoint the key binding targets of individual monomers, on the basis of which structure-based optimization and modification can be carried out to obtain lead molecules with enhanced activity and superior selectivity.

In summary, these four classes of natural monomeric compounds hold tremendous potential as precursor resources for islet function modulators, and subsequent target identification, SAR analysis, and drug-likeness optimization will strongly promote the rational development of novel glucose-lowering drugs based on natural products.

## 5. Stem Cell Therapy: An Ascendant Strategy for Functional β-Cell Restoration

As discussed in the preceding sections, most natural products with direct β-cell-protective effects remain at the preclinical stage, and their clinical translation has been hampered by unresolved issues such as bioavailability, target validation, and formulation standardization. In parallel, stem cell-based therapies have recently emerged as a rapidly advancing area within β-cell replacement research. Because this field directly addresses the restoration of functional β-cell mass and has already reached clinical proof-of-concept, it provides a useful complementary context for the natural product-focused discussion of this review. The following section therefore highlights selected recent advances in stem cell-derived β-cell replacement and their implications for β-cell restoration.

In recent years, stem cell-based therapies have achieved remarkable progress in diabetes treatment, with the core rationale of rebuilding endogenous glucose regulation by supplementing or regenerating functional pancreatic β-cells [156,157,158]. Since both T1DM, in which β-cells are selectively destroyed by autoimmunity, and T2DM, in which β-cells undergo dysfunction and dedifferentiation under chronic metabolic stress, ultimately share a final common pathway of relative or absolute deficiency of functional β-cell mass [157,159], introducing glucose-responsive, insulin-secreting cells from an external source represents a conceptually fundamental therapeutic strategy.

The key technical route is directed differentiation of pluripotent stem cells, but this process remains challenging. Early protocols often produced polyhormonal or functionally immature cells with insufficient glucose-stimulated insulin secretion (GSIS) [156,157]. Stem cell-derived islets are not fully equivalent to primary islets in function, transcriptional identity, or chromatin accessibility, and current protocols still yield only about 40% monohormonal insulin-positive cells, with some cells acquiring an enterochromaffin-like identity and uncontrolled ratios of β, α, and δ cells [160]. The importance of islet cytoarchitecture is underscored by the compromised GSIS of isolated β cells compared with intact islets, which can be partially rescued by pseudo-islet formation with appropriate endocrine and vascular niche cells [161]. Moreover, recapitulating the precise temporal coordination of Nodal/Activin A, WNT, retinoic acid (RA), FGF, BMP, SHH, and Notch pathways remains a fundamental obstacle to reproducing pancreatic organogenesis in vitro [156].

Despite these challenges, current protocols can sequentially differentiate human embryonic stem cells (hESCs) or induced pluripotent stem cells (iPSCs) into definitive endoderm, primitive gut tube, posterior foregut, pancreatic endoderm, and ultimately pancreatic progenitors co-expressing PDX1 and NKX6.1 and even SC-β cells with GSIS function [156,158,162].

These cells have shown therapeutic potential in animal models, reversing hyperglycemia in streptozotocin-induced diabetic immunodeficient mice after subcutaneous macroencapsulation or kidney capsule transplantation [162]. More importantly, this strategy has entered human clinical trials. In Vertex’s Phase I/II trial of a fully differentiated SC-islet product, full-dose recipients restored postprandial C-peptide secretion, achieved HbA1c below 7%, eliminated severe hypoglycemic events, and 10 of 12 participants achieved insulin independence by month 12 [163,164]. Encapsulated hPSC-derived pancreatic progenitors have also been assessed in early-phase trials, although fibrotic responses and limited engraftment have constrained efficacy [165,166]. Critical commentary has emphasized that initial results, while encouraging, still require lifelong immunosuppression and that low C-peptide levels in some trials cannot be definitively attributed to the graft rather than residual endogenous secretion or altered renal clearance [167]. Collectively, these clinical observations—including durable insulin independence in a subset of T1DM patients—provide the first direct evidence that stem cell-derived β-cell replacement can restore endogenous insulin secretion in humans. This stepwise validation from rodents to humans demonstrates that stem cell-derived β-cell replacement has progressed from proof-of-concept to clinical demonstration, providing a practical solution to the donor pancreas shortage [163].

Meanwhile, mesenchymal stem cells (MSCs) provide another paradigm through their immunomodulatory, anti-inflammatory, pro-angiogenic, and anti-apoptotic paracrine functions, protecting and repairing residual endocrine function rather than directly replacing β-cells. Adipose-derived MSC infusion in T2DM rats lowered blood glucose, and the “stem cell educator” strategy has been evaluated in clinical trials, with reports of increased C-peptide levels in T1DM patients and improved insulin sensitivity in T2DM patients [168,169]. MSC-derived exosomes also carry bioactive molecules that alleviate hyperglycemia in T2DM [170]. These findings suggest that stem cell therapy extends beyond simple cell replacement, and early clinical evidence supports its potential as an adjunctive approach, although the evidence remains preliminary.

Despite these promising prospects, a considerable gap remains between clinical demonstration and broad application. The most fundamental barrier is prohibitive cost, driven by weeks of GMP-grade directed differentiation, extensive quality control, and logistics [163,171]. Additionally, the estimated therapeutic dose of about 10^9^ cells per human patient poses substantial scalability hurdles, and cryopreservation of SC-derived islet aggregates remains a critical bottleneck due to low post-thaw viability and non-uniform cooling [160,164]. Human β-cells also have intrinsically low proliferative capacity, further limiting ex vivo expansion [172]. Post-transplant immune rejection and autoimmune recurrence remain unresolved, as autoreactive T-cell memory in T1DM will attack transplanted SC-β cells unless continuous systemic immunosuppression is used, which carries significant toxicity [161,173]. Encapsulation devices, gene-editing strategies, and immunomodulatory approaches are under investigation but have not yet achieved long-term graft survival without systemic immunosuppression. Recent advances, such as engineering SC-β cells to express CD155 or co-aggregating them with regenerative macrophages, have shown promise in improving engraftment and function, but their clinical applicability remains to be established [160,165,173,174]. Thus, although clinical proof-of-concept has been achieved, the current evidence does not yet support the widespread clinical application of stem cell-based β-cell replacement.

Thus, while stem cell therapy offers a potential “functional cure” for selected patients with severe diabetes, its high cost, complex manufacturing, and unresolved immune barriers make it unlikely to become a widely accessible routine treatment in the near term. Therefore, until stem cell therapies achieve broader applicability, natural product interventions that directly improve β-cell function and survival retain irreplaceable practical value, particularly for the large T2DM population, owing to their low cost, convenience, and accessibility. Moreover, insights from stem cell research can inform the screening and evaluation of β-cell-protective natural products, and cell-free strategies such as small extracellular vesicles targeting β-cell senescence further illustrate the convergence between regenerative and pharmacological approaches [175]. Ultimately, stem cell therapies and natural products are complementary rather than competing strategies, serving different patient populations and disease stages.

## 6. Discussion

This review provides a synthesis of the mechanisms by which natural products regulate pancreatic β-cell function. Existing evidence indicates that natural active ingredients influence islet function primarily through three major pathways. First, they promote insulin secretion, involving multiple key steps in the excitation–secretion coupling of pancreatic β cells: these include closing KATP channels to trigger membrane depolarization and subsequent opening of VDCCs to promote calcium influx, mobilizing endoplasmic reticulum calcium stores to release Ca^2+^ via IP_3_ receptor mediation, and modulating the activities of metabolic enzymes such as glucokinase to enhance the ability of cells to sense and metabolize glucose. Second, they indirectly enhance insulin secretion by modulating the incretin system, specifically through inhibiting DPP–IV activity to delay the degradation of endogenous GLP-1 and direct agonism at GLP-1 receptors to activate the downstream cAMP/PKA signaling pathway. Third, they exert comprehensive protection of pancreatic β cells, encompassing multiple levels, including the maintenance of the differentiated phenotype of pancreatic β cells and promotion of regeneration, alleviation of oxidative stress, inhibition of apoptosis and pyroptosis, attenuation of inflammatory responses, and suppression of toxic hIAPP aggregation.

In terms of promoting proliferation and regeneration, Pdx-1, as the core transcription factor governing β-cell development and functional maintenance, is a common target of a variety of natural products; meanwhile, the Notch signaling pathway and epigenetic modifications are also involved. Regarding the alleviation of oxidative stress, natural products can exert effects by enhancing the activities of endogenous antioxidant enzymes such as SOD, GPx, and CAT, activating the Nrf2 signaling pathway, or directly scavenging free radicals. In the context of inhibiting cell death, modulating the Bcl-2/Bax balance and suppressing the caspase cascade are the primary mechanisms for inhibiting apoptosis; inhibiting NLRP3 inflammasome activation can block pyroptosis; and suppressing abnormal hIAPP aggregation and the endoplasmic reticulum stress it induces is also an important pathway for protecting β cells. With respect to attenuating inflammatory responses, inhibiting the activation of pro-inflammatory signaling pathways such as NF-κB, p38 MAPK, and JNK is the core mechanism. Furthermore, some natural products have been demonstrated to have islet-protective or glucose-lowering effects, but their precise molecular targets and signaling networks have not yet been fully elucidated. Collectively, these mechanisms reveal the characteristic of natural products in regulating pancreatic β-cell function through multiple pathways, including promoting insulin secretion, modulating incretins, and providing cytoprotection, thereby offering a theoretical reference for the development of glucose-lowering drugs targeting islet function.

The pathogenesis of T2DM involves two core components: insulin resistance (IR) and pancreatic β-cell dysfunction. Currently used glucose-lowering agents, including oral insulin secretagogues, DPP–IV inhibitors, injectable insulin, and GLP-1 receptor agonists, can effectively control blood glucose; however, their effects are primarily directed at the IR component, with limited improvement of pancreatic β-cell dysfunction. In contrast, natural products from diverse sources exhibit a combination of glucose-lowering effects and islet function restoration, offering a promising avenue to address the limitations of existing drugs in islet function protection. Therefore, natural products can not only serve as potential insulin secretion promoters but, by virtue of their multiple pharmacological activities such as anti-inflammatory, antioxidant, and anti-apoptotic effects, are also expected to delay the progressive decline of islet function in the early stages of T2DM.

Many of the natural products discussed in this review are derived from medicinal plants with a long history of traditional use and relatively well-characterized pharmacological profiles, such as Maidong (*Ophiopogonis Radix*) [70], *Stevia rebaudiana* Bertoni [58], Ginseng [59], *H. rosa-sinensis* L [23,24], *Centaurium erythraea* Rafn [80,81], *Balanites aegyptiaca* [152,153], bitter melon (*Momordica charantia* L.) [26,176], among others. For these plants, both ethnopharmacological experience and modern experimental studies support their potential to modulate islet function and improve glycemic control, suggesting that extracts obtained from such sources may have relatively reliable activity. However, despite this background, the clinical translation of these natural products remains limited. Most of the available evidence is still derived from cell lines, isolated islets, or animal models, and well-designed randomized controlled trials are scarce. This discrepancy between traditional use and clinical development reflects a broader translational gap that has been repeatedly highlighted in the field. Therefore, accelerating the connection between preclinical findings and clinical application has become an urgent priority. Future work should focus on well-characterized extracts and active constituents from these traditional medicinal plants, with particular attention to bioavailability, pharmacokinetics, dose standardization, safety, and herb–drug interactions. Only through such efforts can the therapeutic potential of these natural products be reliably evaluated and ultimately translated into clinically useful interventions.

Although natural products show promising prospects in regulating islet function, their translation to clinical application still faces multiple challenges. First, most natural products have a complex composition, and their defined active ingredients and structure–activity relationships (SAR) have not been fully elucidated, posing difficulties for component standardization and quality control. For example, the ethanolic extract of *Annona squamosa* simultaneously contains three components—rutin, proanthocyanidin, and squafosacin G; although this extract possesses both KATP channel-independent direct insulinotropic activity and DPP–IV inhibitory activity, the relative contribution of each monomer to these activities remains unclear [39,40]. The tuber aqueous extract PTY-2 of *Pueraria tuberosa* exerts antidiabetic effects through the dual mechanisms of DPP–IV inhibition and GLP-1R/GIPR agonism; however, whether this is attributable to a multi-target effect of a single component or the synergistic action of multiple components remains unresolved [41]. Various extracts of *Camellia sinensis* also exhibit the dual activities of closing KATP channels to directly promote insulin secretion and inhibiting DPP–IV, yet their chemical composition is extremely complex, making it difficult to determine whether the source of activity is a single component or multi-component synergy [20,21,22,38]. These numerous studies indicate that difficulties in active ingredient identification and unclear SAR have become prominent bottlenecks in the translation of natural products to standardized drug development.

Second, the oral bioavailability of natural products is generally low, and their metabolic characteristics, tissue distribution, and distribution in target organs in vivo require further in-depth investigation. Moreover, existing studies are mostly limited to preclinical models; although some natural products originate from traditional medicinal systems and have a long history of human use, there is a lack of rigorously designed randomized controlled clinical trial data to support their efficacy and safety in complex physiological environments. Indeed, to date, none of the natural products covered in this review—whether crude extracts, fractions, or purified compounds—have entered clinical trials specifically aimed at β-cell protection or restoration; they remain entirely preclinical, and no clinical efficacy can be claimed for any of these agents at present. Another key issue is that the direct targets on β cells and the upstream and downstream signaling networks regulated by natural products remain unclear, making it difficult to deepen the mechanistic understanding of their actions. Although a few candidates, including Ci leaves extract [43], *Commiphora myrrha* [48], ST3 [126], and PPAG [137], have already been tested in isolated human islets, these studies still represent ex vivo or in vitro experiments and cannot be interpreted as evidence of clinical efficacy. Furthermore, their pharmacokinetic profiles, bioavailability, target validation, and clinical efficacy still require systematic evaluation. The above issues collectively constrain the translation of natural products from basic research to clinical treatment.

In this context, it is useful to briefly contrast the clinical translation status of natural products with that of stem cell-based β-cell replacement, which has recently progressed from preclinical proof-of-concept to clinical demonstration. As detailed in Section 5, transplantation of stem cell-derived β cells has reversed hyperglycemia in diabetic animal models, and early clinical trials have shown that some recipients achieved restored C-peptide secretion and even insulin independence [162,163,164]. However, this strategy still faces considerable barriers, including prohibitive cost, manufacturing complexity, unresolved immune rejection, and limited scalability [160,163,171]. These limitations mean that, despite its clinical proof-of-concept, stem cell therapy is unlikely to become a widely accessible routine treatment in the near term.

Therefore, natural products and stem cell therapies should be regarded as complementary rather than competing approaches. stem cell therapy offers a potential “functional cure” for selected patients with severe diabetes, its high cost, complex manufacturing, and unresolved immune barriers make it unlikely to become a widely accessible routine treatment in the near term. Therefore, until stem cell therapies achieve broader applicability, natural product interventions that directly improve β-cell function and survival retain irreplaceable practical value, particularly for the large T2DM population, owing to their low cost, convenience, and accessibility. Moreover, insights from stem cell research can inform the screening and evaluation of β-cell-protective natural products, and cell-free strategies such as small extracellular vesicles targeting β-cell senescence further illustrate the convergence between regenerative and pharmacological approaches [175]. Ultimately, stem cell therapies and natural products are complementary rather than competing strategies, serving different patient populations and disease stages.

## 7. Conclusions

This review summarizes the multi-pathway regulation of pancreatic β-cell function by natural products, including promotion of insulin secretion, modulation of the incretin system, and cytoprotection, thereby providing abundant lead compounds and a mechanistic framework for developing agents that combine glycemic control with β-cell protection. However, the natural products covered remain largely at the pre-clinical stage, with unresolved structure–activity relationships and with most identified targets confined to known regulatory pathways. In parallel, stem cell-based β-cell replacement has already entered clinical trials and demonstrated the potential to restore endogenous insulin secretion in patients with T1DM, but its broad application is limited by prohibitive costs, complex manufacturing, and unresolved immune barriers. These two strategies should therefore be viewed as complementary: stem cell therapy offers a potential cure for patients with severe β-cell deficiency, whereas natural products provide a more accessible approach for early intervention and functional maintenance in the broader T2DM population. Future research should prioritize target validation, development of standardized formulations, pharmacokinetic characterization, safety evaluation, and well-designed clinical trials to assess β-cell protection and restoration in patients with T2DM. The coordinated development of both approaches will contribute to a more integrated framework for restoring and preserving β-cell function in diabetes.

## Figures and Tables

**Table 1:** Comparative positioning of the current review relative to selected recent representative reviews on natural products and pancreatic β-cell function.

Review Feature	Semwal et al., 2021 [7]	Xu et al., 2018 [8]	Ríos et al., 2015 [9]	This Work
Primary Focus	β-cell protection and regeneration	Anti-T2DM activities and overall metabolic regulation	Antidiabetic mechanisms and clinical trials of medicinal plants	Direct regulation of β-cell function and survival
Data Scope/Evidence Sources	Specific molecules (e.g., berberine, curcumin) and plant extracts; mainly in vitro/animal data	Major signaling pathways (PPAR, AMPK, PI3K/AKT, etc.); multiple metabolic targets	Broad range of antidiabetic mechanisms; multiple medicinal plants; clinical trials	Insulin secretion pathways (KATP–VDCC, IP_3_/ER Ca^2+^); incretin system; β-cell protection (Pdx-1, Notch, Nrf2, NLRP3, hIAPP/ER stress); target identification tools
Mechanistic Depth	Mainly phenotypic; molecular mechanisms largely unclear	Pathway-level; overall metabolism-focused	Broad but general; limited β-cell-specific detail	Specific molecular events; calcium signaling; epigenetic modifications
Coverage of β-Cell Toxicopathological Mechanisms	Limited to apoptosis/regeneration; no hIAPP, NLRP3, or ER stress	Not covered	Not covered	Systematically covers hIAPP aggregation, NLRP3 pyroptosis, and ER stress
Key Unresolved Issues	Mechanism; bioavailability; metabolomics	Drug vs. supplement; toxicity; standardization; clinical validation	Drug interactions; clinical evidence	Active constituent attribution; SAR; single- vs. multi-component effects; bioavailability; direct targets; clinical validation
Core Contribution	β-cell regeneration as a drug discovery strategy	Useful data and information for further NP research	Broad reference framework for antidiabetic NPs	Multi-pathway mechanism framework; leads for combined glycemic control and β-cell protection

**Table 2:** Multifaceted regulatory effects of natural products on pancreatic β cells.

Category	Natural Products	Mechanism	Refs
Other direct insulinotropic effects	The leaves extract of *Costus igneus*	Upregulates glucokinase activity, downregulates glucose-6-phosphatase activity, enhances insulin and GLUT2 gene expression, and improves glucose sensing and metabolism in β cells.	[43]
	Thymoquinone	Depletes NAD(P)H and generates H_2_O_2_, alters the intracellular redox state, increases the glucose-dependent ATP/ADP ratio, and directly enhances insulin secretion.	[44]
	*Commiphora myrrha*	Directly activates signaling pathways associated with excitation–secretion coupling in β cells, thereby rapidly promoting insulin release.	[48]
	Coixol	Dose-dependently enhances glucose-stimulated insulin secretion in isolated islets and MIN-6 cells; the insulinotropic effect is more pronounced under high-glucose conditions, without significant cytotoxicity.	[46]
	Steviophethanoside	Significantly promotes insulin secretion in INS-1 cells with low toxicity; reported for the first time as a natural phenylpropanoid insulinotropic compound.	[47]
Maintenance of the differentiated phenotype and promotion of regeneration of pancreatic β cells	The leaf extract of *Cichorium intybus*	Induces the differentiation of P19 embryonal carcinoma stem cells into insulin-secreting cells, suggesting the potential to promote β-cell neogenesis.	[49]
	The ethyl acetate fraction of *Curcuma amada* rhizome extract	Exerts antidiabetic effects at the genomic level by regulating signal transduction of glucose- and apoptosis-related genes; stimulates β-cell regeneration and increases insulin secretion.	[50]
	Ellagic acid (EA)	Stimulates glucose-induced insulin secretion, improves pancreatic β-cell morphology and number, and restores the structural integrity of islets.	[51]
	The hydroalcoholic extract of the *Hibiscus rosa-sinensis* flower	Improves glycemic control without lowering normal blood glucose levels, and exhibits antidiabetic activity with a favorable safety profile in animal models.	[52]
Alleviation of oxidative stress	Silibinin	Upregulates ERα, activates the Nrf2 pathway, increases HO-1 and SOD2 expression, reduces ROS generation, and enhances β-cell survival and insulin secretion.	[53]
	The whole seeds and cold-pressed seed oil of *Nigella sativa*	Increases the activities of antioxidant enzymes such as SOD and GPx, reduces MDA levels, protects pancreatic tissue, decreases β-cell apoptosis, and partially promotes β-cell regeneration.	[54,55,56]
	The extract of *Polysiphonia japonica*	Alleviates palmitic acid-induced oxidative stress and improves β-cell function by inhibiting BMP signaling.	[57]
	The aqueous extract of *Stevia rebaudiana*	Increases SOD and CAT activities, reverses STZ- or alloxan-induced oxidative damage, reduces blood glucose, and elevates insulin levels.	[58]
	Ginseng oligopeptides	Attenuates inflammation by inhibiting NF-κB expression, ameliorates oxidative stress, and inhibits β-cell apoptosis by modulating Bcl-2 family expression.	[59]
	Madecassoside	Enhances antioxidant status, inhibits lipid peroxidation, improves pancreatic β-cell structure and function, and elevates plasma insulin levels.	[60]
	The extract of *Leea macrophylla* root	Improves the histopathological morphology of islets and promotes the repair and protection of β cells after STZ-induced injury.	[61]
	The seed oil of *Citrullus lanatus*	Activates the Akt/GSK-3β/Nrf2 pathway, upregulates antioxidant protein expression, repairs islet tissue damage, and elevates plasma insulin levels.	[16]
	The ethanolic extract of *Quercus liaotungensis* Koidz	Inhibits the p38 MAPK signaling pathway, activates the Nrf2 pathway, protects pancreatic β-cell function, and elevates serum insulin levels.	[62]
	Betulin	Reduces blood glucose and glycated hemoglobin levels, exerts antioxidant effects to counteract oxidative stress, and increases islet area and the proportion of insulin-positive β cells.	[63]
Inhibition of β-cell apoptosis and pyroptosis	The ethanolic extract of *Morus alba*’s leaves	Activates the AMPK/mTOR signaling pathway, induces protective autophagy, and inhibits β-cell apoptosis.	[64]
	The high-molecular-weight fraction of *Gymnema sylvestre*	Inhibits caspase-3/7 activity triggered by inflammatory factors; upregulates casein kinase II to regulate ER stress; enhances endogenous antioxidant defense; and suppresses β-cell apoptosis at multiple levels.	[65]
	Andrographolide	Suppresses the upregulation of TXNIP expression, blocks NLRP3 inflammasome activation, and alleviates cigarette smoke-induced pancreatic β-cell pyroptosis.	[66,67,68]
Attenuation of inflammatory responses	Cocoa powder and Carob flour	Inhibits the NF-κB signaling pathway, downregulates IL-6 and TNF-α levels, and attenuates pancreatic inflammation.	[69]
	The polysaccharide extract of *Ophiopogon japonicus* (L. f.) Ker Gawl.	Suppresses the IKK–NF-κB axis, reduces IL-1β expression and NF-κB p65 nuclear translocation, and alleviates islet inflammation.	[70]
	The ethanolic extract of *Artemisia dracunculus* L.	Inhibits p38 MAPK phosphorylation, attenuates IL-1β-mediated NF-κB activity, and downregulates the expression of inflammatory genes.	[71]
	The ethanolic extract of Clinacanthus nutans’ leaves	Protects pancreatic β cells from apoptosis by modulating oxidative stress and inflammatory responses, enhancing antioxidant enzyme activities, and inhibiting the JNK pathway and caspase-3 activation.	[72]
Mechanisms not yet fully elucidated	The seed extract of *Berberis orthobotrys*	Reduces blood glucose, total cholesterol, and triglyceride levels; histological analysis reveals protection of islet architecture, and the activity may be mediated by its major metabolite berberine, which could regulate insulin release or promote β-cell repair.	[73]
	The ethanolic extract of *Balanites aegyptiaca*	Its chitosan nanoparticles (BA Ex-CS NPs) scavenge ROS, exert antioxidant and anti-inflammatory effects, promote pancreatic β-cell regeneration and neogenesis, increase insulin secretion, reduce blood glucose, and improve hepatic and renal function.	[74]

Note: This table summarizes the natural products and their mechanisms of action that are not covered in Figure 3, Figure 4, Figure 5, Figure 6, Figure 7 and Figure 8 of the main text, serving as a supplement to the illustrated content.

**Table 3:** Monomeric compounds with regulatory effects on pancreatic β-cell function.

Category	Name	Structure	Mechanism	Refs
Flavonoids	Rutin	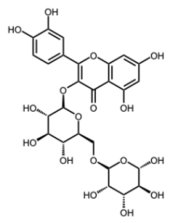	Modulating the incretin system	[40]
	Proanthocyanidins	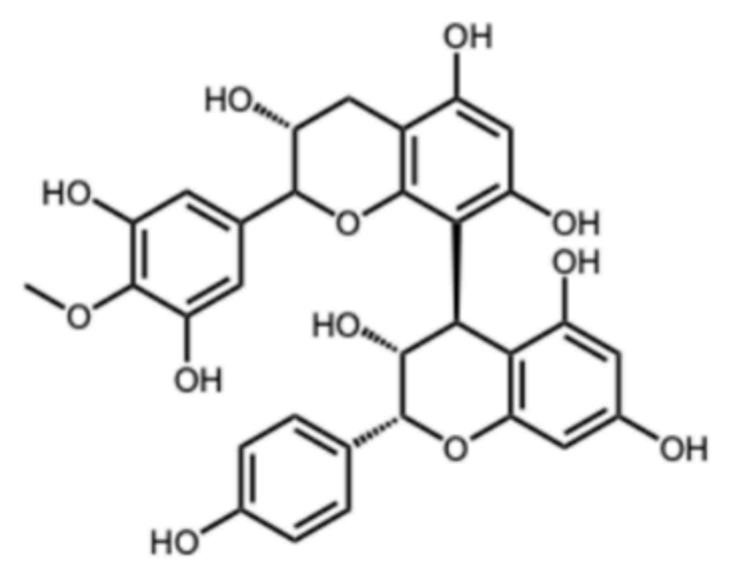	Modulating the incretin system	[40]
	Epigallocatechin-3-gallate	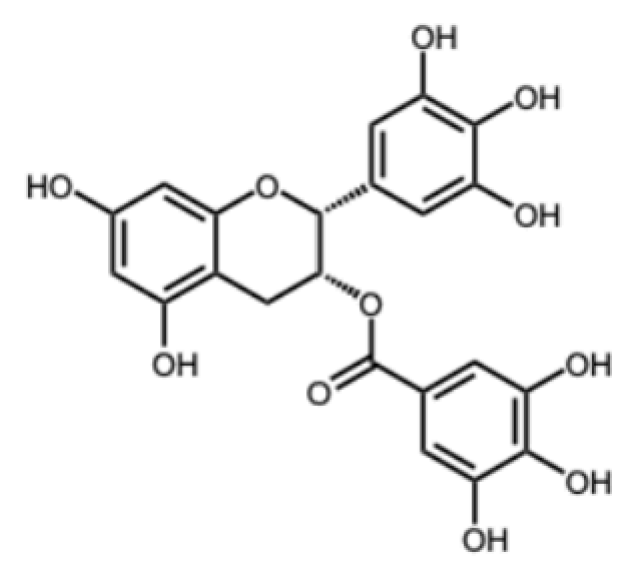	β-cell regeneration	[94,95]
	Vitexin	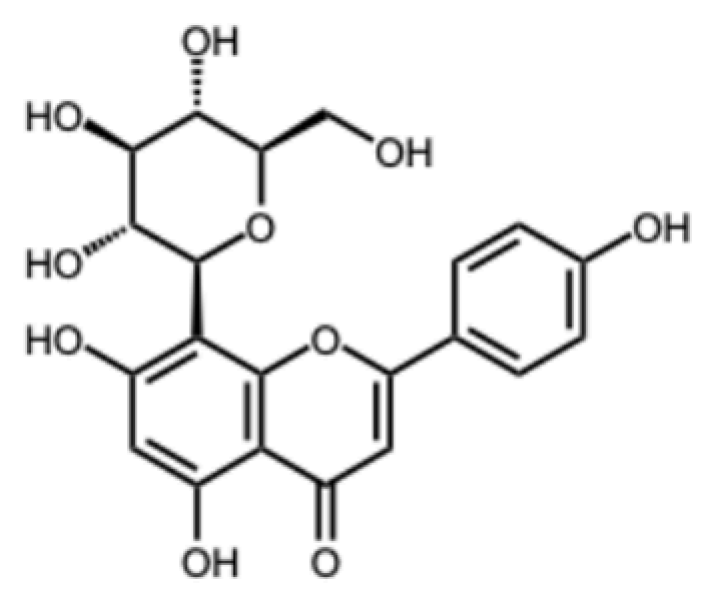	Antioxidative stress	[108]
	Silibinin	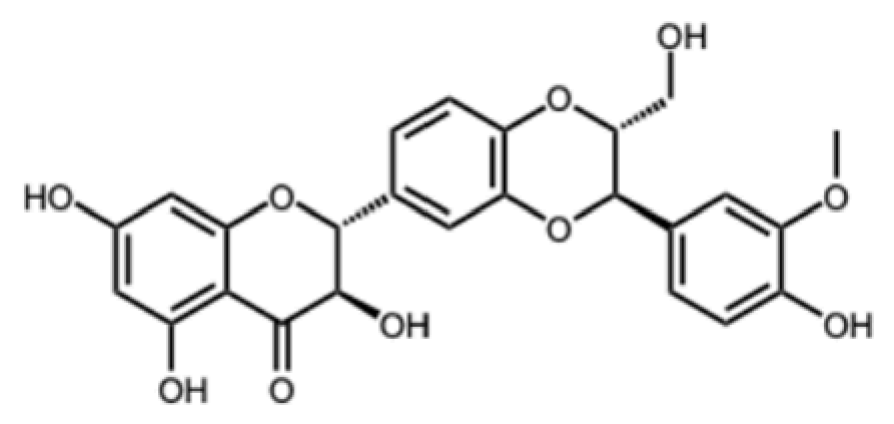	Antioxidative stress	[53]
	Luteolin	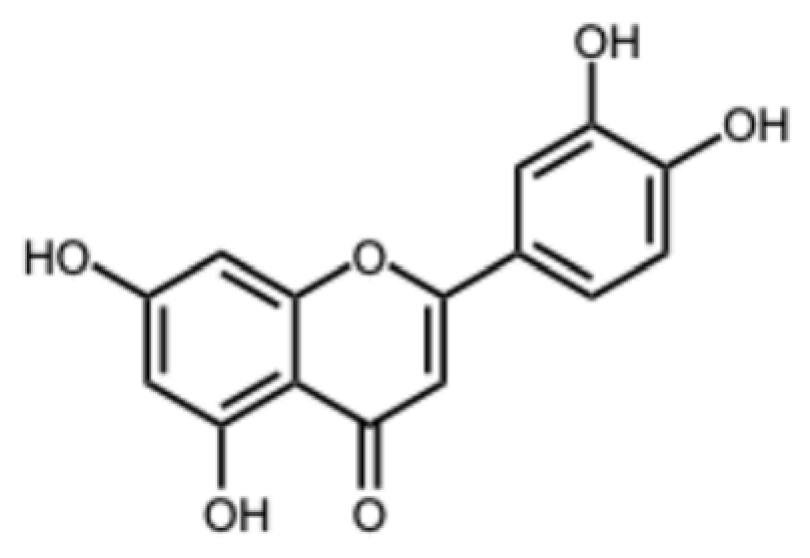	Antioxidative stress	[124]
	The polyphenolic flavonoid myricetin	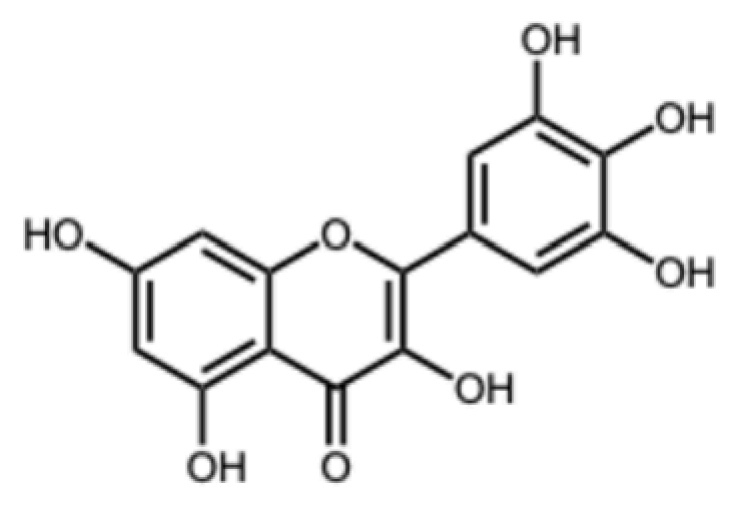	Anti-apoptosis	[145]
Terpenoids	Boschnaloside	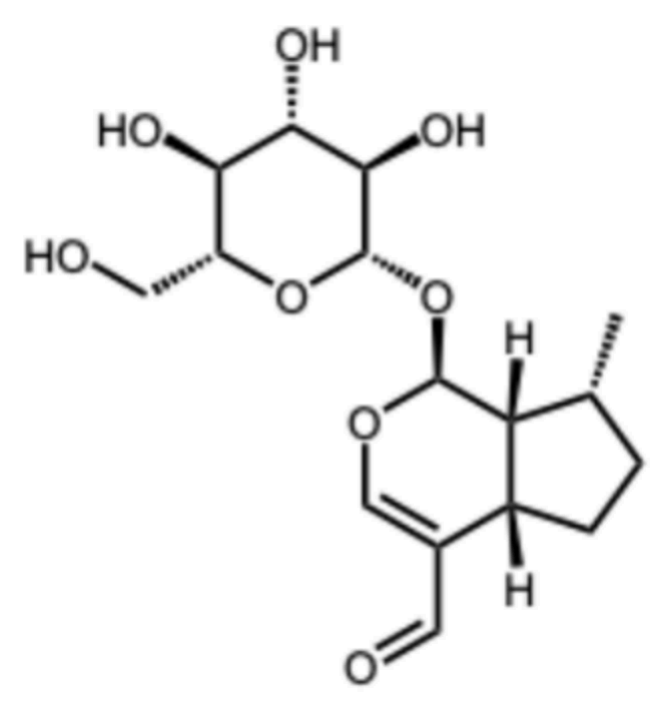	β-cell regeneration	[92]
	Madecassoside	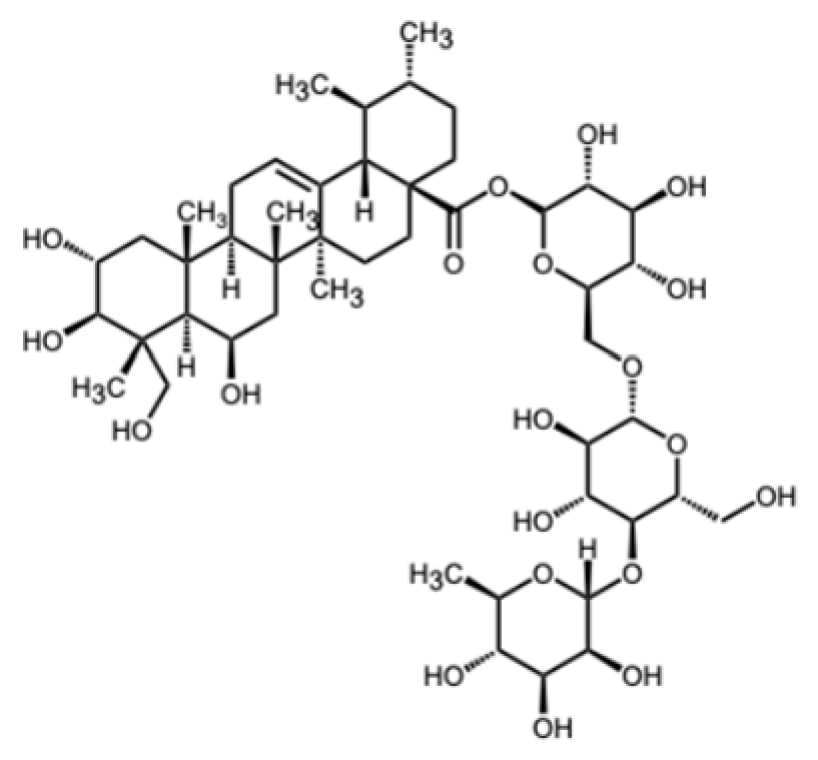	Antioxidative stress	[60]
	Betulin	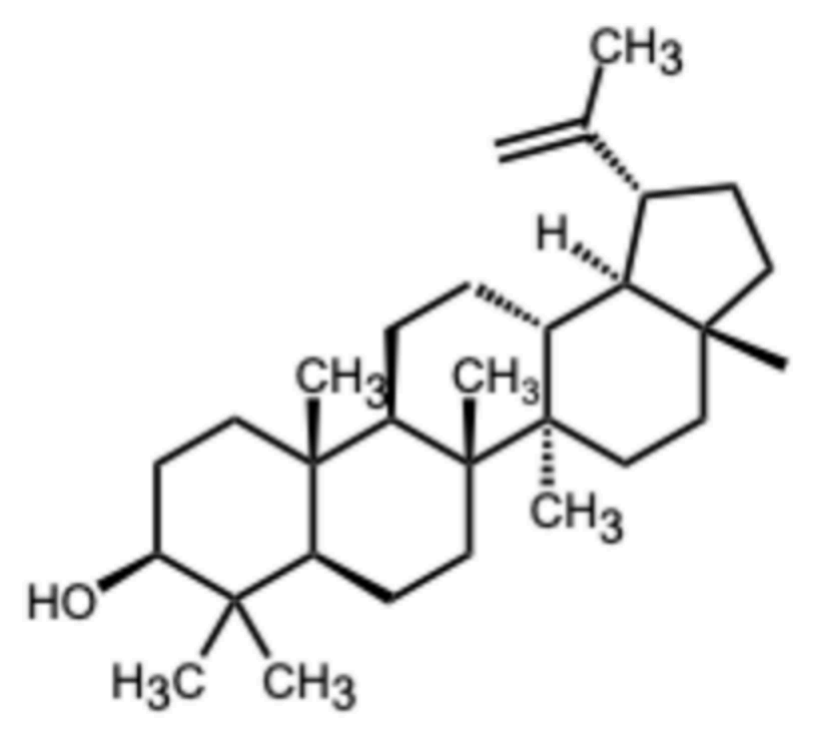	Antioxidative stress	[63]
	Andrographolide	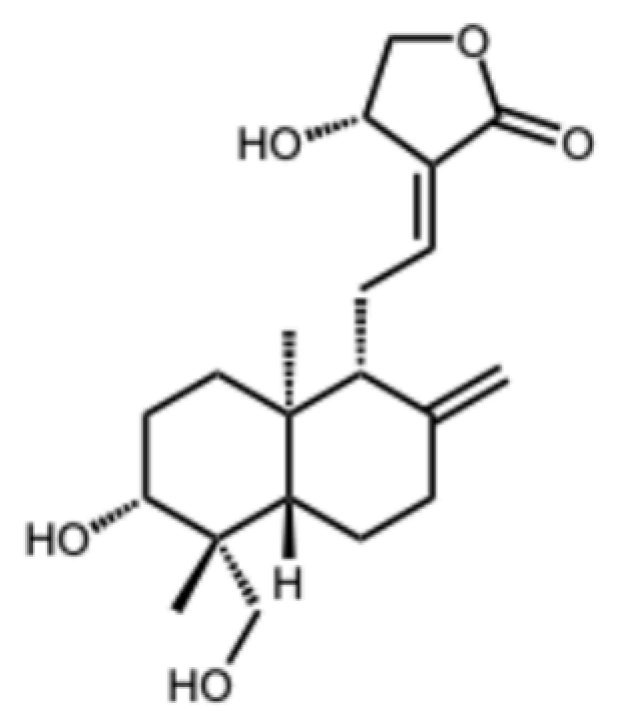	Anti-pyroptosis	[66,67,68,155]
Steroids	Withaferin A	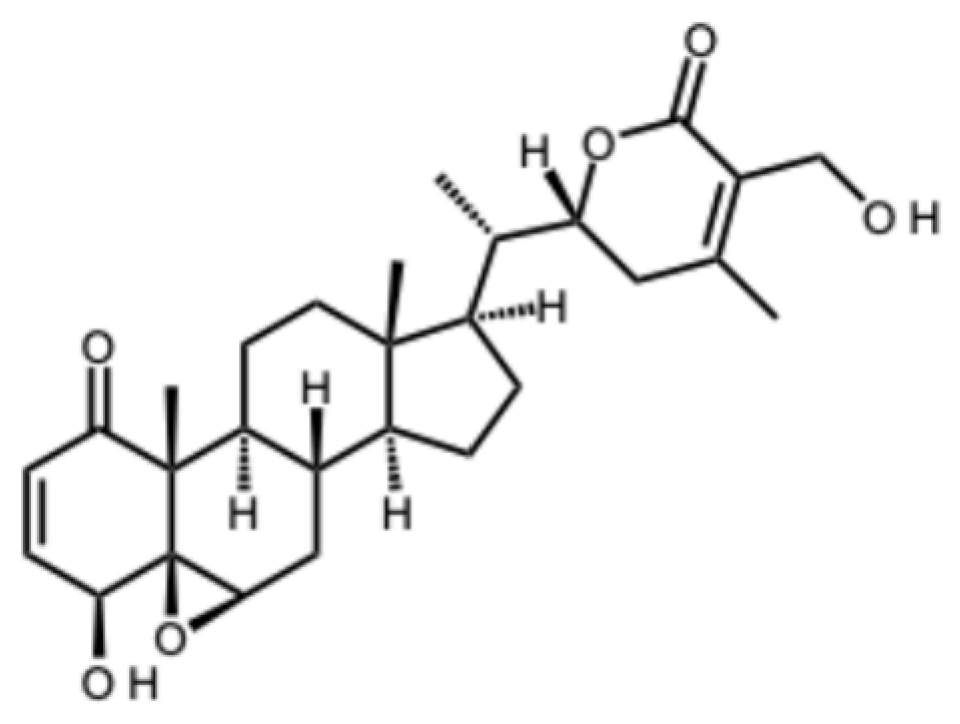	Anti-apoptosis	[139]
	Withacoagulin	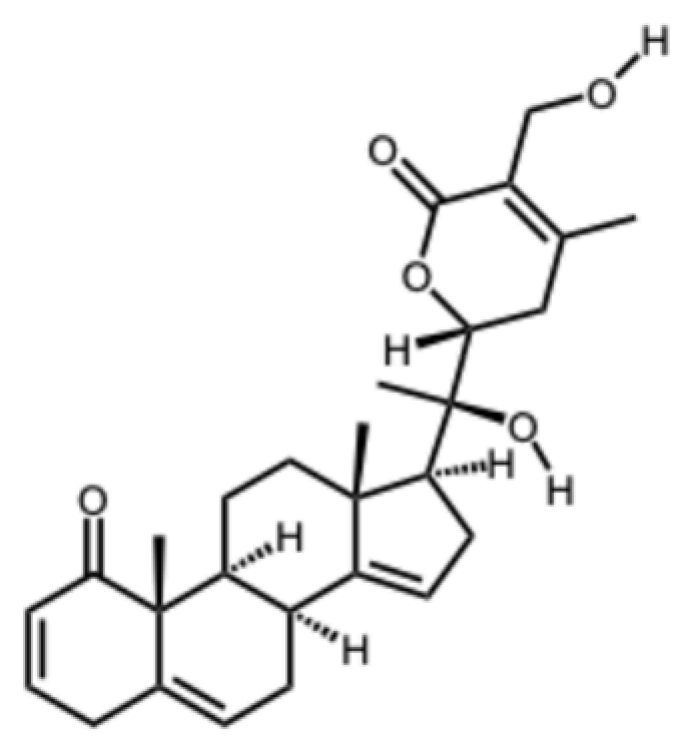	Anti-apoptosis	[139]
Organic acids	Quinic acid	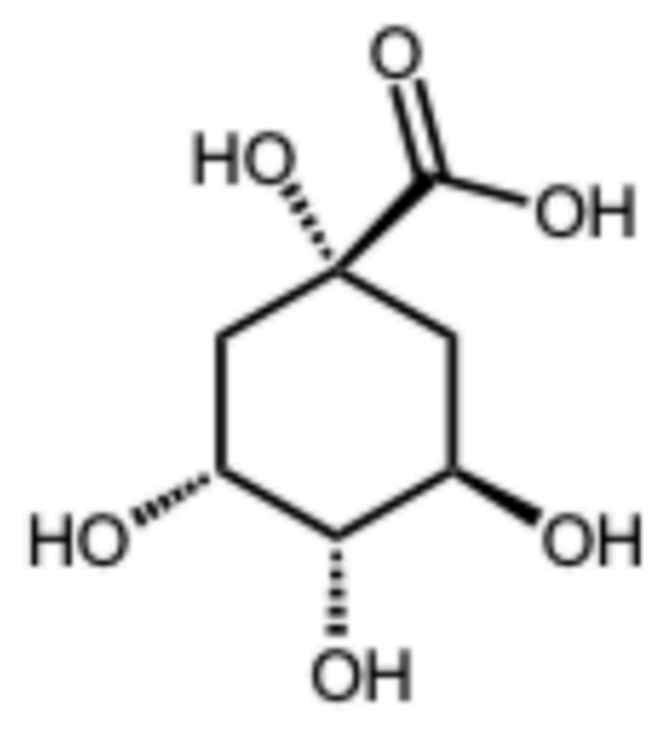	Calcium mobilization	[34]
Alkaloids	Coixol	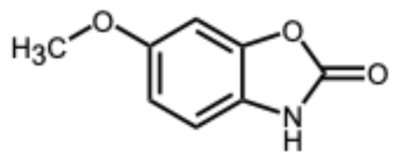	Insulin secretion	[46]
Phenylpropanoids	Steviophethanoside	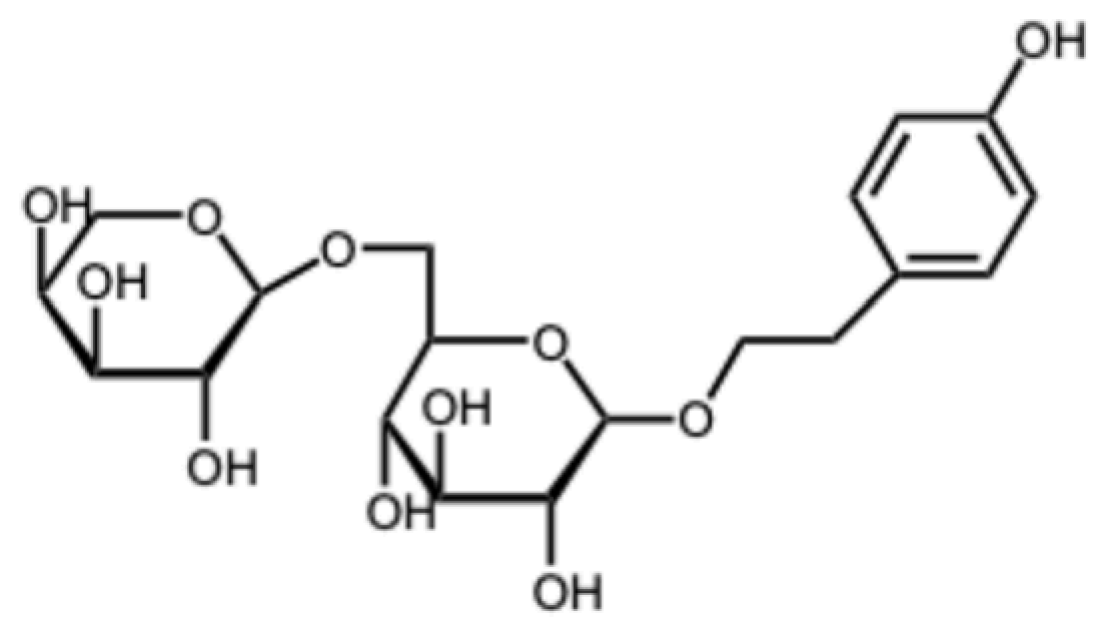	Insulin secretion	[47]
Polyketides	Squafosacin G	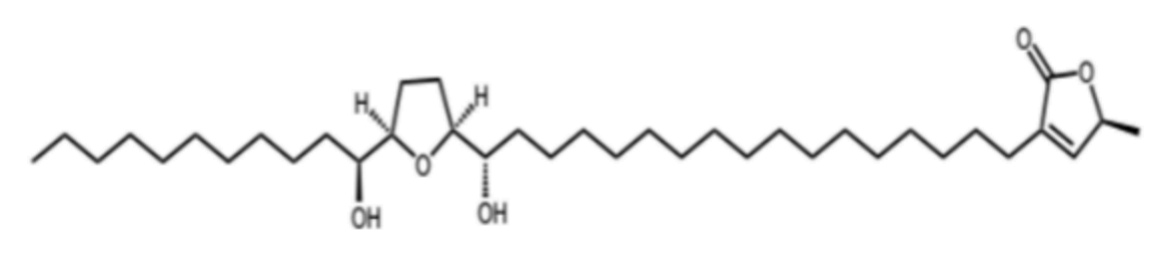	Incretin modulation	[40]
Phenolic acids	Ellagic acid	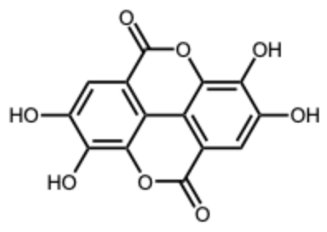	β-cell regeneration	[51]

## Data Availability

No new data were created or analyzed in this study. Data sharing is not applicable to this article.

## Abbreviations

T2DM	Type 2 Diabetes Mellitus
IR	Insulin Resistance
DPP–IV	Dipeptidyl Peptidase IV
SGLT-2	Sodium-Glucose Cotransporter 2
GLP-1	Glucagon-Like Peptide-1
hIAPP	Human Islet Amyloid Polypeptide
NCDs	Noncommunicable Diseases
MetS	Metabolic Syndrome
GSIS	Glucose-Stimulated Insulin Secretion
KATP channels	ATP-Sensitive Potassium Channels
VDCCs	Voltage-Dependent Calcium Channels
IP_3_	1,4,5-Trisphosphate
ER	Endoplasmic Reticulum
PLC	Phospholipase C
PIP_2_	Phosphatidylinositol 4,5-Bisphosphate
GIP	Glucose-Dependent Insulinotropic Polypeptide
GLUT2	Glucose Transporter 2
Bcl-2	B-cell Lymphoma 2
cAMP/PKA/CREB	Cyclic Adenosine Monophosphate/Protein Kinase A/cAMP-Response Element Binding Protein
G-6-pase	Glucose-6-Phosphatase
NAD(P)H	Nicotinamide Adenine Dinucleotide Phosphate
Pdx-1	Pancreatic Duodenal Homeobox Factor-1
Nrf2	Nuclear Factor Erythroid 2-Related Factor 2
ROS	Reactive Oxygen Species
Bax	Bcl-2/BCL-2-Associated X Protein
NLRP3	NOD-Like Receptor Thermal Protein Domain Associated Protein 3
NF-Κb	Nuclear Factor Kappa-Light-Chain-Enhancer of Activated B Cells
DLL4	Delta-Like Ligand 4; Hes1, Hes Family BHLH Transcription Factor 1
SOD	Superoxide Dismutase; GPx, Glutathione Peroxidase
CAT	Catalase
GSH	Glutathione
MDA	Malondialdehyde
STZ	Streptozotocin
Sp1	Specificity Protein 1
PA	Palmitic Acid
UCP-2	Uncoupling Protein-2
HO-1	Heme Oxygenase-1
BMP	Bone Morphogenetic Protein
JNK	c-Jun N-Terminal Kinase
IL-1β	Interleukin-1β
OM	Oligomannuronate
TXNIP	Thioredoxin Interacting Protein
TNF-α	Tumor Necrosis Factor-α
p38 MAPK	p38 Mitogen-Activated Protein Kinase
IL-6	Interleukin-6
IRS-1	Insulin Receptor Substrate 1
CMC	Cell Membrane Chromatography
SPR	Surface Plasmon Resonance
RA	Retinoic Acid
hESCs	Human Embryonic Stem Cells
iPSCs	Induced Pluripotent Stem Cells
MSCs	Mesenchymal Stem Cells
